# Does improved storage technology promote modern input use and food security? Evidence from a randomized trial in Uganda

**DOI:** 10.1016/j.jdeveco.2018.07.006

**Published:** 2018-11

**Authors:** Oluwatoba J. Omotilewa, Jacob Ricker-Gilbert, John Herbert Ainembabazi, Gerald E. Shively

**Affiliations:** a Department of Agricultural Economics, Purdue University, West Lafayette, IN 47907, USA; b Development Economics Data Group, World Bank, 1818 H Street NW, Washington, DC 20433, USA; c International Institute of Tropical Agriculture (IITA), P.O. Box 7878, Kampala, Uganda; d Alliance for a Green Revolution in Africa (AGRA), P.O. Box 66773, Nairobi, Kenya; e Purdue Policy Research Institute, Purdue University, West Lafayette, IN 47907, USA

**Keywords:** Post-harvest storage losses, Improved storage technology, RCT, Higher-yielding maize adoption, PICS hermetic bags, Uganda

## Abstract

We use panel data from a randomized controlled trial (RCT) administered among 1200 smallholders in Uganda to evaluate input use and food security impacts of an improved maize storage technology. After two seasons, households who received the technology were 10 percentage points more likely to plant hybrid maize varieties that are more susceptible to insect pests in storage than traditional lower-yielding varieties. Treated smallholders also stored maize for a longer period, reported a substantial drop in storage losses, and were less likely to use storage chemicals than untreated cohorts. Our results indicate that policies to promote soft kernel high-yielding hybrid maize varieties in sub-Saharan Africa should consider an improvement in post-harvest storage as a complementary intervention to increase adoption of these varieties.

## Highlights

• The higher-yielding hybrid maize varieties often promoted in SSA store poorly.
• We assess the impacts of improved storage technology on planting of these varieties.
• Improved storage technology increases the cultivation of these varieties.
• The storage technology increases storage length and reduces storage losses.
• Improved storage should complement promotion of higher-yielding maize varieties.

## 1 Introduction

Many poverty alleviation and development programs implemented in sub-Saharan Africa (SSA) focus on increasing agricultural production and smallholder productivity, frequently by encouraging smallholders to increase their use of improved seed varieties and chemical fertilizer (Evenson and Gollin, 2003; Pingali, 2012). Often, however, these programs ignore what happens to output in the post-harvest season (World Bank, 2011). This is problematic, because while maize is the most important staple food in Eastern and Southern Africa, the softer kernel high-yielding hybrid varieties commonly promoted there offer less natural protection to insect attacks during storage compared with the lower-yielding traditional varieties that store relatively well (Golob, 2002; Smale et al., 1995). As a result, smallholders face a dilemma. Should they plant high-yielding varieties that carry storage risks or traditional varieties with lower yields, but less vulnerability to insect attacks during storage (Ricker-Gilbert and Jones, 2015)?

In this study, we use a randomized controlled trial (RCT) to measure whether a smallholder's ability to store maize using an improved storage technology affects the household's storage decision and, ultimately, its subsequent decisions about using modern inputs. In our RCT, we provided to a randomly selected group of households one Purdue Improved Crop Storage (PICS) hermetic (airtight) storage bag—an improved grain storage technology—that eliminates insect pests in storage when properly sealed. We compare choices and decisions among this treated group against a control group, consisting of farmers that received no intervention and continued to use traditional storage techniques. Because not all households who were randomly offered the technology chose to use it, we estimate intention-to-treat (ITT) effects for its policy relevance. Moreover, the impacts on treated households who took-up the offer and actually used the storage technology are likely to be larger. That is, unlike the local average treatment effects (LATE) on compliant households, the estimated ITT effects average impacts across both treated households who used the technology and those who did not.^1^

The present article has two main objectives. First, we estimate whether receipt of an improved storage technology leads to input-related behavioral changes in maize cultivation. The behavioral changes of interest include the uptake of improved maize varieties in terms of adoption and intensity (share of area planted to improved varieties), and, possibly, the use of inorganic fertilizer for increasing maize yields. Because improved maize varieties are more susceptible to pest attacks during storage due to their softer kernels and open husks relative to the traditional, lower-yielding varieties, farmers face an increased post-harvest storage risk when choosing to plant these improved varieties. Using panel data from Ethiopia, Dercon and Christiaensen (2011) showed that ex-post production risk (rainfall variability) reduces a household's adoption of improved inputs (inorganic fertilizer) ex-ante. If the same holds true in our context, when households have the ability to store improved maize varieties in an effective, chemical-free hermetic storage technology, their storage risks or storability concerns may be mitigated. Thus, access to hermetic storage technology may influence the cultivation of improved maize varieties.^2^ Further evidence that storability concerns may negatively influence the adoption of improved maize varieties comes from Malawi (Katengeza et al., 2012; Lunduka et al., 2012), Zimbabwe (Derera et al., 2006), and Uganda (Obaa et al., 2005) where farmers expressed preference for traditional varieties due to storability concerns.

Our second objective is to explore some of the possible channels through which receipt of an improved storage technology may influence the adoption of improved maize varieties. For example, these include (i) the quantity of maize stored at harvest, (ii) the duration of time that maize is stored, and (iii) use of chemical insecticides, often referred to as storage chemicals, on stored maize. We also examine the impact of the technology on the percentage of self-reported post-harvest losses (PHL) indicated by households.^3^ Previous studies show that hermetic storage technologies are effective at limiting maize damage in storage (De Groote et al., 2013; Njoroge et al., 2014; Tefera et al., 2011). Therefore, one might reasonably expect access to an improved storage method to influence storage decisions.

To our knowledge, few published findings explore the causal link between storage technology and inputs use among smallholder farmers in SSA. Furthermore, there has been little or no rigorous impact analysis thus far for hermetic storage bags in SSA, as discussed in a recent review of the topic (Sheahan and Barrett, 2017). With few exceptions, issues relating to post-harvest losses have not been considered in studies that evaluate the adoption of improved inputs such as seed and inorganic fertilizer among smallholder farm households. Thus, the relationships between post-harvest management practices, storability concerns, and adoption of improved seed varieties in SSA remain poorly understood. Understanding these relationships is important for future maize productivity and food security in the region (Bezu et al., 2014; Mason and Smale, 2013).

The present article makes two main contributions to the literature. First, we fill a policy research gap for SSA by estimating a causal relationship between improved storage technology and improved input adoption. Ricker-Gilbert and Jones (2015) examined this linkage using observational panel data from Malawi, and found the use of chemical insecticides to be significantly associated with the probability of adopting improved seed varieties. However, the authors stop short of concluding causal impact in their study, and advocate for the use of an RCT to answer the question more fully in the future. Our impact evaluation with experimental design complements and builds upon Ricker-Gilbert and Jones' (2015) study.

The majority of studies that have estimated the impacts of improved storage technologies in developing countries are observational. For instance, Gitonga et al. (2013) used propensity score matching (PSM) to evaluate the economic and food security impacts of hermetic metal silo on duration of maize storage, loss abatement, and spending on storage chemicals for maize-growing farmers in Kenya. In Central America, Bokusheva et al. (2012) used regression analysis and a Tobit model to estimate impacts of hermetic metal silo on adopter's well-being, sales of production, and the number of months a farmer purchased foods, respectively.

To our knowledge, our study is one of a very few to have evaluated improved storage technologies as part of an RCT. Ndegwa et al. (2016) used RCT to investigate the effectiveness of hermetic storage bags at reducing storage losses and its economic viability in an on-farm trial in one district of Kenya. Basu and Wong (2015) conducted an evaluation of a randomized seasonal food storage and food credit programs or treatments in West Timor Indonesia. They investigated whether access to improved storage technology helps households to transfer assets (staple food endowment) from harvest to lean season, smoothing inter-seasonal household consumption. They find that the storage treatment increased non-food consumption but had no effect on staple food consumption. In a more recent study, Aggarwal et al. (2017) experimentally evaluate a group-based grain storage scheme through savings clubs in Kenya. They find that individuals who joined the group-based savings clubs were more likely to store maize to be consumed or sold at least one month after harvest. Our study builds on this sparse literature by testing if there is a behavioral link on the part of smallholders between improved storage technology, storage decisions and input adoption decisions the next season.

Our second contribution is to use a large sample (nearly 1200 smallholders) surveyed over two years (2014 and 2016). The experimental panel dataset has a broad geographic scope that gives it a semblance of being nationally representative of maize producing households in Uganda. The broader geographic scope relative to previous studies that evaluate improved storage technologies confers a measure of external validity on our study to support the internal validity offered by our experimental design. As such, our results should be generalizable to similar populations elsewhere in SSA.

Results from our study indicate that households treated with the improved storage technology are 10 percentage points more likely to plant hybrid maize seed varieties the following year (significant with p-value<0.05), consistent with observational findings reported by Ricker-Gilbert and Jones (2015) in Malawi. Our findings have implications for improved maize variety adoption, maize productivity, and potentially, food security among smallholder households; because they suggest that, an improved storage technology can be a complementary intervention for promoting the adoption of improved maize varieties.

On the possible channels of impact, we find that the treated households who received the technology do not increase the quantity of maize stored at harvest, likely because maize is their staple crop so they adopt a safety-first mentality and used the improved hermetic bag in place of a traditional bag. However, treated households store maize with the intent of consuming it for three weeks longer (significant with p-value <0.01), and they store maize with the intention of selling it for one week longer (significant with p-value <0.10). In addition, treated households are less likely to use chemical insecticides on stored maize (significant with p-value <0.05). These findings are consistent with Gitonga et al. (2013). Lastly, we find that the treated households report storage losses 2.2 to 2.5 percentage points lower than control households (significant with p-value<0.05). These indicates that between 65 and 71 percent of the average reported losses in stored maize can be eliminated with an improved storage technology.

## 2 Maize production and post-harvest storage losses in Uganda

### 2.1 Maize production

Maize is one of the major staple foods in Uganda and the most important cereal crop. It is produced mostly for household subsistence, but it is evolving as a cash crop, and the Government of Uganda believes that smallholder farmers can increase their maize productivity by adopting improved technologies such as hybrids and open pollinated varieties (Ugandan Bureau of Statistics (UBoS), 2007). According to UBoS, annual maize production is estimated at 1.5 million metric tons, and 90 percent of this is used for human consumption with the remaining 10 percent for animal feeds.

Average maize yields are estimated at 1.5 metric tons per hectare among smallholders (Matsumoto and Yamano, 2011; Okoboi, 2010). Despite the development and release of improved varieties, yields remain low partly due to low uptake of these varieties, fake seeds on the market (Bold et al., 2015) and low input use. Evidence suggests that any increase in maize production is due to area expansion, rather than an increase in productivity (Kasenge et al., 2001; Okoboi, 2010; Sserunkuuma, 2005). In general, the use of improved agricultural technologies remains low in Uganda relative to other countries in SSA, and it is commonly believed that increased uptake of these inputs will increase productivity. For instance, Matsumoto and Yamano (2011) sampled 895 households from 94 rural local council one administrative units across Uganda and found that only 3 percent used inorganic fertilizer, with an average application rate of 2.4 kg/ha. Sheahan and Barrett (2014) found that only 36% of maize farmers in Uganda bought improved seeds for cultivation; and at 3.2 percent, Uganda had the lowest proportion of inorganic fertilizer users among six SSA countries examined in their study.

### 2.2 Post-harvest storage losses in maize

Uganda is located along the equator, so high temperature and relative humidity create a suitable environment for insect pests to attack maize in storage (Tefera, 2012). Nevertheless, precise quantitative assessment of storage losses in Uganda is difficult due to high year-on-year variability in pest infestation (Costa, 2015); and magnitudes of loss vary depending on the measures used to assess the losses (Affognon et al., 2015), length of storage, or type of maize stored (FAO, 2003).

Using self-reported measures from a nationally representative dataset from the World Bank's Living Standard Measurement Study—Integrated Surveys on Agriculture (LSMS-ISA), Kaminski and Christiaensen (2014) estimated on-farm storage losses for maize to be 3.9 percent on average in Uganda.^4^ Although these self-reported losses appear low on average, they are concentrated and the magnitude can reach up to 100 percent (total loss) for some households (Kaminski and Christiaensen, 2014).^5^ In addition, anecdotal evidence suggests that Ugandan farmers are taking adaptive measures to keep their storage losses low. Such measures include selling entire maize gardens or fields to traders prior to harvest, selling harvested maize immediately or shortly after harvest, and using storage chemicals and other crop protection methods such as spraying or smoking.^6^ These practices could explain why smallholders report low storage losses in general.

### 2.3 Maize storage technologies

Ugandans use multiple maize storage technologies and practices. Table 1 shows the percentage of households using each storage technology or technique prior to our intervention. The predominant technology is the single-layer woven polypropylene bag popularly called “kavera” locally; these bags were used by 71 percent of our sample. Heaping maize in the house, where households leave maize cobs on bare floor was used by 11 percent of households. Other technologies include granaries at 8 percent, and private off-farm facilities at 2 percent. All the above technologies are broadly categorized as traditional storage technologies. The total use of hermetic (airtight) improved storage technology was less than 1 percent in our sample at baseline. Kaminski and Christiaensen (2014), using LSMS-ISA data, also reported that the use of improved storage technology was generally low at 0.6 percent in Uganda. Farmers in SSA generally lack access to improved storage technologies to store bumper harvests, and Uganda is no exception (Costa, 2015).

**Table 1:** Proportion of smallholder households using each storage technology at baseline.

Storage Technology	Season 1, 2014 (%)	Season 2, 2013 (%)	Sample Average (%)
Woven polypropylene bag	71.2	70.5	70.9
Heaped in House	10.7	10.7	10.7
Traditional granaries	6.5	7.3	6.9
Private off-farm store	1.8	1.9	1.8
Improved granaries	1.2	0.8	1.0
Open-air hanging	0.8	0.9	0.9
Hermetic (drum/silo/jerry can)	0.8	0.6	0.7
Metal silo/drum	0.2	0.2	0.2
Hermetic bags	0.1	0.2	0.1
Community storage facility	0.1	0.1	0.1
Others	6.7	6.7	6.7
Observations	1146	1076	1111

Conversely, farmers regularly use chemical insecticides on maize stored in traditional technologies to prevent on-farm storage pest attacks, but these chemicals are toxic if not used properly, and largely unregulated (Williamson et al., 2008). At baseline, 11 percent of households in our sample had applied chemical insecticides on stored maize. In this context, chemical-free hermetic technologies can be a safe and effective alternative. For instance, the PICS technology given to farmers in our intervention is an airtight triple-layered technology consisting of two high-density polyethylene inner liners and one outer layer of woven polypropylene bag. It works by impeding oxygen diffusion from outside the bag to its interior. Thus, when storage insect pests lack oxygen for metabolism, they become inactive, desiccate and die (Murdock et al., 2012).

On-going efforts seek to promote the use of hermetic storage bags under the PICS—phase III (PICS3) project in Uganda. As such, the data collected in our intervention provides us with a platform to evaluate the impacts of the technology using field experiments (the intervention is discussed in detail in the next section). Uganda makes for an interesting case study because, as shown in Table 1, the use of hermetic storage technologies among smallholders is practically non-existent and we can conduct a rigorous impact evaluation of the technology, which has been largely missing in SSA (Sheahan and Barrett, 2017).

## 3 Data, sampling and experimental design

### 3.1 Data

The data for this study come from two rounds of household-level experimental panel survey. The baseline survey was conducted from October to December 2014, followed by the PICS3 intervention in July 2015 (discussed in section 3.3), and the follow-up survey occurred in 2016 during the same months as in the baseline survey. The baseline survey covered two cropping cycles: the second agricultural season of 2013 (September 2013–January 2014) and the first agricultural season of 2014 (March–August 2014). The post-intervention survey also covered two cropping cycles: the second agricultural season of 2015 (September 2015–January 2016) and the first agricultural season of 2016 (March–August 2016).

We used a structured, pre-tested questionnaire that includes modules on household demographic characteristics and production-related details such as total area of cultivated land, area cultivated per crop, input use levels, and crop yields. The survey tool also asks about grain storage technologies and practices used, and quantities of maize stored at harvest; marketing activities in both harvest and lean periods; assets and household well-being indicators like crop and off-farm income; gender differentiated questions on decision making at household levels; food and nutrition security questions; and social networks.

### 3.2 Sampling

To select the study area and representative households in our sample, we used a multi-level stratified sampling approach. First, we identified the major maize producing districts across Uganda using previous data from the publicly available LSMS-ISA dataset. Then based on production volume, we purposely selected two districts in each of the four regions—Central without Kampala, Eastern, Western, and Northern—across Uganda to give the sampling a semblance of nationally representative maize-growing smallholders in Uganda. Kampala region, which is largely urban, was excluded. Second, within each selected district, we further purposely selected three major maize producing sub-counties with assistance from the district agricultural/production officers (DAOs).

Afterwards, we included three levels of randomization in our sampling process. First, we randomly selected two parishes in each sub-county and followed the parish selection with another random selection of one local council one (LC1) per parish.^7^ Lastly, we randomly selected twenty-five households that we interviewed from each LC1. The LC1 chairpersons or leaders provided lists of village residents to facilitate the random selection of the households at the LC1 level. We assigned a number to each name on the list and randomly chose twenty-five using a computer random number generator. In total, we sampled 1200 households (25 per LC1 in 48 LC1s). Following data cleanup, 1190 valid household responses remained.

### 3.3 Experimental design/Intervention

**Fig. 1:**
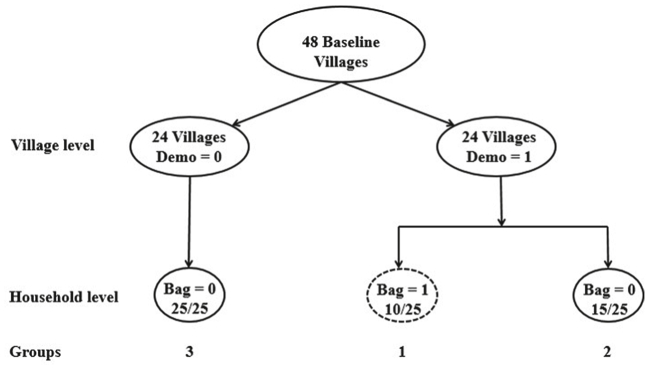
Experimental design.

After the baseline survey in 2014, we conducted two randomized interventions in 2015. The first occurred at the village level, and provided information about the use and effectiveness of the hermetic bags. We randomly divided the 48 LC1s into two equal groups of 24. Within each sub-county, we randomly selected one LC1 into a demonstration group, and another into a non-demonstration group (see Fig. 1). Between July and August of 2015, a non-governmental organization (NGO) called Cooperative League of the USA (CLUSA) in Uganda implemented demonstration activities within the demonstration villages, to create awareness about the improved storage technology. These villages received demonstrations in which participants who attended were introduced to the technology and instructed on how to use it correctly. All households in the demonstration villages were invited to attend these activities regardless of whether they were sampled (as part of the study) or not. We refer to the demonstration and non-demonstration LC1s as DEMO and non-DEMO LC1s, respectively.

The second treatment occurred at the household level, and we implemented it to measure the impacts of actually receiving one hermetic bag that can store up to 100 kg of shelled maize on the outcomes of interest in this study. It occurred shortly after CLUSA had implemented the village demonstration activities in 2015. Within the DEMO LC1s, we gave one hermetic bag per household to 10 households. These were randomly selected from the 25 households that were initially sampled from the baseline survey. Therefore, eligibility for the second treatment was conditional on living within a village that received a demonstration about the technology. For the second treatment, the choice of a sub-sample of 10 households (out of twenty-five) per treatment village was based on power calculations to arrive at a minimum detectable effect (MDE) of 0.2 SD in outcomes between the treated and control groups at the household level.^8^ Overall, 240 exogenously treated households received a hermetic bag in our sample. We refer to them as treated households.

In summary, our study has three groups of households: treated households within DEMO LC1s (group 1); exposed households who were untreated within the DEMO LC1s (group 2); and lastly, untreated group of households in non-DEMO LC1s (group 3) (see Fig. 1 for more details). It is possible that not all randomly treated households attended the demonstration activities in DEMO LC1s, but trained technicians who implemented the second treatment trained the households at the point of giving out the bags.

### 3.4 Testing for potential attrition bias

Two years after conducting our baseline survey and after two complete harvest cycles following our treatment intervention, we returned for the post-intervention survey. From the 1190 households in our baseline sample, we were able to re-interview 1146 indicating a 96% rate of resampling. In addition, 233 of the 240 treated households were successfully re-interviewed. The attrition rate is less than 3%. Relative to other studies in the region, our attrition rates are comparable or lower (e.g., Matsumoto and Yamano, 2011). In general, the main cause of attrition was households migrating out of the area.

However, to test for the presence of attrition bias in our sample, following Hidrobo et al. (2014) and suggestion by Duflo et al. (2007), we regressed our outcome variables and other covariates on a binary indicator equal to one for attritted households, and zero otherwise. We found no systematic difference between attritted and returning households for all outcome variables (See Table A.1 in Appendix A). For other covariates, with the exception of age, we also did not find any evidence that attritted households are systematically different from returning households. Generally, attritted households are six years younger, on average, indicating mobility among younger households. Very low attrition rates among the treated households and in the entire sample, coupled with failure to find significant difference in our simple mean tests, suggests attrition bias is likely not an issue in our study.^9^

## 4 Causal pathways

In this study, we make a few but likely realistic assumptions about how storage technology affects future household decisions. First, we assume that households believe improved maize varieties have higher yields than traditional varieties. Second, storability of the improved maize varieties is a concern for households when compared to traditional varieties. Third, treated households believe that improved storage technologies, such as hermetic bags, can effectively store maize with limited concerns. These assumptions are supported by anecdotal evidence and published literature (Derera et al., 2006; Katengeza et al., 2012; Ntege-Nanyeenya et al., 1997).

Consider a rational household's decision on whether to plant improved higher-yielding maize varieties with storability concerns, to increase production vs. the low-yielding traditional maize varieties that are less susceptible to storage pest attacks. Storing improved maize varieties for later use (sale or consumption) in the absence of an effective storage technology may result in significant losses. Therefore, if a household expects higher storage losses from cultivating improved varieties, such household will plant less of the varieties. With an improved storage technology, however, smallholders can effectively store maize for consumption through the lean period, reducing food expenditure. Alternatively, they can effectively store to take advantage of inter-temporal price arbitrage by selling in the lean period.

Therefore, we posit that if households have access to an improved storage technology to overcome storability concerns associated with the high-yielding maize varieties in the post-harvest season, they should store better quality maize for a longer period for either consumption or sale and cultivate more of the improved seed varieties. Because the use of a hermetic storage technology should increase confidence in households' ability to store maize effectively, households may be encouraged to cultivate the improved varieties that would otherwise store poorly in traditional storage technologies.

## 5 Empirical framework

We focus on estimating the direct impacts of randomly assigned improved storage technology on household input use and storage behaviors. To estimate these impacts, we compare potential outcomes for treated households with the potential outcomes in the absence of the treatment. Typically, only one outcome can be observed for any individual at a time and it is not possible to identify the counterfactual outcome. However, with randomization, we can obtain the average potential outcome for the control group, which is a valid counterfactual devoid of selection bias (Angrist and Pischke, 2009; Duflo et al., 2007; Wooldridge, 2010).

Furthermore, because we have the benefit of observing each household in our sample before and after treatment, we employ three different estimators to estimate our *Intention-to-Treat* (ITT) effects (τ). These estimators are i) simple means difference (SMD) using only our post-intervention data, ii) difference-in-difference (DiD) using baseline and post-intervention data, and iii) household fixed effects (FE) using baseline and post-intervention data. From the expected causal pathways described above, we expect the ITT estimates of our treatment effect on the use of higher-yielding maize varieties to be positive.^10^ We estimate all binary dependent variables via linear probability model (LPM).

### 5.1 Intention-to-treat (ITT)

For the first specification (SMD), we estimate the ITT effect for household i in LC1 j and region r as follows:

(1) $$y_{ijr}=\;\lambda +\tau_{SMD}T_{i}+\beta X_{\mathrm{ijr}}+\sigma_{r}+\;\varepsilon_{ijr}$$

Let $y_{ijr}$ be the observed outcome variables (improved seed use; share of maize land cultivated to improved variety; fertilizer use; quantity stored; length of storage for consumption and sale; and storage chemical use); and $T_{i}$ is a household-level indicator that equals one if the household was randomly offered a hermetic bag (treatment) and zero otherwise. In addition, $X_{\mathrm{ijr}}$ is a vector of household characteristics such as age, education status of the household head, household size, sex of household head and family size, which are included for precision. Lastly, $\sigma_{r}$ is the region fixed-effects to account for variations across the regions, and $\varepsilon_{ijr}$ is the idiosyncratic error term. The coefficient on the treatment assignment term, $\tau_{SMD}$, captures the average effect of being randomly offered the improved storage technology in a DEMO village, and it is the ITT parameter of interest. The coefficient β is a vector of parameters associated with household characteristics.

The second estimator is a pooled (DiD) estimator for the households in time *t*. It is estimated as follows:

(2) $$y_{ijrt}=\;\lambda +\;\varphi T_{i}+\kappa S_{t}+\tau_{DiD}S_{t}*T_{i}+\beta X_{\mathrm{ijr}}+\sigma_{r}+\;\varepsilon_{ijrt}$$

In addition to the variables described in equation (1), $S_{t}$ represents a time fixed-effect that is equal to one if the observations is from the 2016 post-treatment survey and zero otherwise, and $S_{t}*T_{i}$ is the interaction between the two variables. Our parameter of interest is $\tau_{DiD}$, which is the ITT using the pooled difference-in-differences. Any correlation between the time-constant unobserved household-level heterogeneity embedded in $\varepsilon_{ijrt}$ and the ITT is removed through DiD estimation (Cameron and Trivedi, 2005; Wooldridge, 2010).

Finally, we use the household FE estimator as follows:

(3) $$y_{ijt}=\;\lambda +\;\tau_{FE}T_{i}+\beta X_{\mathrm{ijt}}+\theta_{t}+\mu_{i}+\;\varepsilon_{ijt}$$

In this case, $\tau_{FE}$ gives us the ITT estimate using the fixed-effects (within) estimator; $\theta_{t}$ is the time categorical variable for agricultural seasons, and $\mu_{i}$ is the time-constant household unobserved heterogeneity. The FE models $\mu_{i}$ as a parameter to be estimated. Empirically the difference between DiD and FE is that the former removes correlation between the ITT and $\mu_{i}$ (embedded in $\varepsilon_{ijrt}$), while the latter removes correlation between all covariates and $\mu_{i}$ (Wooldridge, 2010). Given that our randomization should take care of both observed and unobserved household-level heterogeneity, the ITT for DiD and FE should be very similar.

In comparing SMD to DiD and FE estimators, though randomization should allow simple mean difference estimation to provide unbiased results of ITT of receiving the technology, the DiD and FE estimators are preferred because they exploit the panel nature of our data. Both estimators remove bias in the second period comparisons between treatment and control groups that could be because of a permanent difference between the two groups. It also removes bias from comparison over time in the treatment group. Lastly, both DiD and FE estimates should add increased robustness to our results, as they are able to remove any time-constant unobserved heterogeneity that is correlated with ITT from our model.

### 5.2 Sampling weights

We used a multi-level stratified sampling approach as earlier described. We sampled 25 households per village irrespective of the village population. With this sampling approach, different households have different probabilities of being sampled and assuming equal probability could lead to biased estimates of the population effects (Cameron and Trivedi, 2005). In our regressions, we use sampling weights that are inversely proportional to the probability of being sampled. The sample weights are calculated as the inverse of 25 (sample size per LC1)/total household population for each LC1. That is, the inverse probability of being selected in any given LC1 for each observation.

Furthermore, in our stratification, since our ultimate sampling units (households) are clustered within a higher or intermediate sampling unit (LC1), we cannot rule out serial correlation within the LC1 clusters. Although the intra-cluster correlation coefficients (ICC) for outcome variables are relatively low, we use heteroscedasticity-robust standard errors, clustered at the LC1 level, for all inference.^11^

### 5.3 Take-up, local average treatment effect (LATE)

Before presenting treatment effects on treated households, we examine actual usage of the technology among households in our sample. First, among treated households who were randomly offered the bags, about 70 percent used the technology to store grains. The main reason for not using the technology was poor harvest due to drought in the seasons following our intervention. Second, for exposed (eligible but not treated) households in the DEMO villages, only 12 percent had used the technology indicating households either bought the bag or received it as gift from a treated neighbor.^12^ Lastly, among households in the non-DEMO villages (pure control group), just about 4.5 percent had taken up the technology post-intervention. These households could have accessed the technology from other sources outside of study as other vendors market the product.

As indicated above, because not all households randomly offered the technology used it, we used the random treatment assignment as an instrument for actual usage of the technology to estimate LATE on actual usage. We discuss and compare this result with the ITT in the results and discussion section.

### 5.4 Multiple hypothesis testing

Whenever there are a large number of measured outcomes, significant coefficients may emerge by chance, even when there are no treatment effects on the outcomes. This multiple inference concern is well known to literature (e.g., Anderson, 2008; Romano and Wolf, 2005; Williams et al., 1999). Fortunately, there are methods to correct standard errors for multiple hypotheses testing without a substantial loss of power as in the Bonferroni correction (Anderson, 2008; Benjamini et al., 2006). Following Ksoll et al. (2016) who used sharpened q-values as implemented in Anderson (2008), we conducted multiple hypotheses correction testing and present adjusted sharpened q-values for our results in Appendix Table A.4.^13^ Our findings are robust across specifications in terms of statistical significance when we adjusted standard errors for multiple hypotheses testing.

## 6 Results and discussion

Before assessing the impact of the intervention, we investigate the success of the randomization process. Thereafter, we present the main results on input use, examine whether the intervention actually changed storage behaviors and reported losses as speculated in our causal pathways, and perform robustness checks on the main results.

### 6.1 Baseline randomization balance checks

Table 2 presents the pre-treatment balance of our baseline randomization. Column (1) shows the mean variables for the control group, column (2) shows the standard deviation, and column (3) shows the regression coefficient of the treatment assignment variable. That is, column (3) shows the ex-ante difference in means between the treated and control variables. In column (4), we present the p-values for statistical inference. Column (5) shows the sample size for each variable.

**Table 2:** Baseline characteristics and balance between treatment and control groups.

Variables	Control		Treated		
	Mean	SD	Coeff.	p-value	N
	(1)	(2)	(3)	(4)	(5)
*Panel A: Dependent Variables*					
=1 if HH planted improved maize seed	0.34	0.475	−0.030	0.404	2235
Share of improved maize area (%)	33.98	47.203	−3.422	0.344	2234
=1 if HH used inorganic fertilizer	0.09	0.287	0.019	0.402	2231
Quantity stored (kg)	606	1024	32.203	0.736	2364
Length of storage for consumption (weeks)	14.4	9.63	−0.729	0.522	2364
Length of storage for sales (weeks)	4.4	6.08	−0.187	0.592	2364
=1 if HH used storage chemical on maize	0.12	0.322	−0.031*	0.078	2362
Self-reported post-harvest losses (%)	3.15	5.99	0.481	0.365	2131
*Panel B: Household Characteristics*					
Age of household head (years)	44.49	14.822	1.188	0.210	2380
Household size	6.35	2.972	0.195	0.446	2380
=1 if female-headed household	0.16	0.367	0.014	0.550	2380
=1 if Polygamous	0.17	0.374	0.007	0.825	2380
=1 if HH head has any form of education	0.89	0.319	0.002	0.932	2380
=1 if HH has radio	0.78	0.416	−0.009	0.766	2370
=1 if HH has mobile phone	0.69	0.463	0.002	0.967	2370
*Panel C: Production and PH practices*					
Total maize area (ha.)	0.53	0.499	−0.013	0.757	2235
Total quantity harvested-maize (kg)	928	1246	−31	0.784	2235
=1 if Traditional storage technology use	0.83	0.382	0.006	0.845	2364
=1 if other improved storage tech. use	0.12	0.110	−0.004	0.558	2364
=1 if hermetic storage technology use	0.009	0.097	−0.007**	0.013	2364
*Panel D: Region Effects*					
=1 if region is Eastern	0.25	0.43	−0.002	0.974	2380
=1 if region is Northern	0.25	0.43	−0.001	0.995	2380
=1 if region is Western	0.25	0.43	0.001	0.995	2380
=1 if region is Central w/o Kampala	0.25	0.43	0.003	0.973	2380

Notes: Columns 1 and 2 report means and standard deviations for control group at baseline. Columns 3 through 5 report results from an OLS regression comparing households in the treatment and control groups in the baseline controlling for region effects and clustering standard errors at the village level. Columns 3 and 4 report the OLS coefficient and p-value corresponding to the binary treatment indicator and column 5 reports the sample size for each regression. ***p < 0.01, **p < 0.05, *p < 0.1.

On average, the use of improved maize varieties, share of maize area cultivated to improved varieties, and inorganic fertilizer use are well balanced between the treatment and control groups, ex-ante. Thirty-four percent sampled households planted improved maize varieties in the control group and there is no significant difference compared to the treatment group. Likewise, the share of area planted to improved seeds is 34 percent in the control group but not different from the treatment group. About 10 percent of the sample used inorganic fertilizer and there is also no significant difference between both groups.

Overall, on the remaining dependent variables, the treatment group stored 32 kg more maize but with no statistically significant difference. Likewise, there are no statistical differences in the length of storage for consumption and sales between both groups of households, ex-ante. For self-reported storage losses, there is also no difference between both groups of households, ex-ante. There is, however, a marginally significant difference between the treatment and control groups for storage chemical use (p-value = 0.078). Members of the treatment group are 3 percentage points less likely to use storage chemicals on their maize than the control group on average. The implication is that our post-intervention estimate of treatment effects for the treatment group may be upward biased if we use only the post-intervention (cross-sectional) data to estimate treatment effect. However, we take care of this bias by pooling both baseline and post-intervention data to form a panel and using the DiD and FE estimators.

On the covariates, the household characteristics such as age and education status of household head, household size, and proxies for access to information such as possession of radio set or mobile phone are all balanced between the two groups. Likewise, the production details and post-harvest storage technologies used are balanced with the exception of hermetic storage technology use. The difference in hermetic storage technology balance is because less than 1 percent of our sample had used the technology at baseline. The difference is statistically significant, but in terms of magnitude, it is not different from zero. Lastly, both groups of households are evenly distributed across the regions in our study area. Overall, our balance check suggests that the randomization process was effective.

We examine the ITT impacts below. Columns (1–2) present the simple mean difference estimates, columns (3–4) present DiD estimates and columns (5–6) present the FE parameter estimates. The first column for each estimator is without covariates; the second column adds covariates.

### 6.2 Improved maize variety adoption

Table 3 presents the ITT effects of a smallholder household being randomly offered an improved storage technology (one hermetic bag that holds 100 kg of shelled maize) on its decisions to plant improved maize varieties the next season. These estimates test whether treated households, who are able to preserve their maize efficiently in the post-harvest period, are subsequently more likely to cultivate higher-yielding varieties of maize that are known to be susceptible to insect pest attacks in storage.

**Table 3:** Treatment effects on households planting improved maize varieties.

Dependent variable:=1 if HH planted improved maize	(1)	(2)	(3)	(4)	(5)	(6)
	SMD	SMD	DiD	DiD	FE	FE
Treatment effect (τ)	0.097** (0.042)	0.095** (0.043)	0.099** (0.048)	0.101** (0.049)	0.099** (0.045)	0.093** (0.046)
Household size		0.012*** (0.004)		0.007*** (0.002)		−0.010* (0.005)
Age of household head		−0.000 (0.001)		−0.001* (0.001)		−0.001 (0.001)
=1 if HH head is educated		0.036 (0.057)		0.095*** (0.034)		0.027 (0.036)
Female headed household		−0.067** (0.031)		−0.092*** (0.030)		0.120* (0.066)
=1 if Eastern Region	0.032 (0.077)	0.019 (0.078)	0.053 (0.061)	0.041 (0.058)		
=1 if Western Region	−0.142** (0.064)	−0.154** (0.063)	−0.097 (0.059)	−0.115** (0.056)		
=1 if Northern Region (0/1)	−0.077 (0.059)	−0.090 (0.060)	−0.043 (0.053)	−0.068 (0.051)		
Season indicators	No	Yes	No	No	Yes	Yes
HH fixed effects?	No	No	No	No	Yes	Yes
Constant	0.277*** (0.059)	0.212** (0.087)	0.341*** (0.047)	0.338*** (0.071)	0.349*** (0.013)	0.399*** (0.087)
Observations	2247	2247	4482	4482	4482	4482
R^2^	0.035	0.052	0.025	0.049	0.030	0.035
Number of households					1245	1245

Notes: For simplicity, the first row shows the ITT estimates for all estimators with and without covariates. Columns (1) and (2) are the simple mean difference estimates ($\tau_{SMD}$); columns (3) and (4) are the DID treatment effect estimates $\left(\tau_{DID}\right)$, which is the ‘treated*post-intervention’ interaction term; and columns (5) and (6) are the treatment effects estimates from the FE estimator $\left(\tau_{FE}\right)$. Robust standard errors, clustered at the LC1 level, are shown in parentheses. ***p < 0.01, **p < 0.05, *p < 0.1.

In columns (1) and (2), we show the simple mean difference estimates without and with covariates, respectively. Both show a similar positive and significant effect. On average, randomly treated households are 9.5–9.7 percentage points more likely to plant improved maize varieties. The similarity in estimates with and without covariates lends some confidence to their stability and consistency.

The DiD estimate presented in column (3) shows that a random offer of improved storage technology increases the likelihood of planting improved maize seed variety by 9.9 percentage points. With the addition of covariates in column (4), the DiD estimate shows that treated households are 10 percentage points more likely to cultivate improved varieties. The FE estimates in columns (5–6) also show a similar positive effect of close to a 10 percentage point increase. These estimates are consistent across all columns, with and without covariates, and are statistically significant at p-value<0.05.

Upon further examination of the treatment groups ex-ante and ex-post, we found that on average, more households in the control group stopped cultivating (“disadopted”) hybrid maize varieties post-intervention. This finding suggests that households who lacked effective storage technology to store the easily susceptible hybrid varieties over a long period stopped cultivating it. Conversely, the level of hybrid seeds remained at the same level for the treatment group who had access to better storage technology.

### 6.3 Share of area planted to improved seed varieties

Table 4 presents the ITT effects of a randomly offered 100 kg hermetic storage bag on the share of area cultivated to improved maize varieties. Similar to the decision to plant improved maize varieties in Table 3, columns (1) and (2) show there is significant effect for the treatment group using the SMD estimator. On average, treated households cultivate a higher share of improved seed varieties by about 9.5 percentage points (significant at p-value <0.05), with or without additional covariates, respectively. From our preferred and more precise DiD and FE estimators, columns (3) through (6) show a higher and more significant magnitude; between 9.7 and 10.4 percentage points marginal increase in share of area planted to improved varieties by treated households, on average. We conclude that improved storage technology increases share of area planted to high-yielding varieties.

**Table 4:** Treatment effects on share of area planted to improved varieties.

Dependent variable: Share of improved maize area (%)	(1)	(2)	(3)	(4)	(5)	(6)
	SMD	SMD	DiD	DiD	FE	FE
Treatment effect (τ)	9.486** (4.011)	9.373** (4.068)	10.250** (4.727)	10.411** (4.739)	10.291** (4.345)	9.727** (4.490)
Household size		1.156*** (0.405)		0.672*** (0.228)		−1.045** (0.519)
Age of household head		−0.033 (0.073)		−0.138 (0.084)		−0.069 (0.117)
=1 if HH head is educated		2.775 (5.644)		8.852** (3.349)		2.732 (3.563)
Female headed household		−6.764** (2.997)		−9.140*** (2.979)		11.376* (6.423)
=1 if Eastern Region	3.402 (7.521)	2.100 (7.547)	5.409 (6.008)	4.224 (5.725)		
=1 if Western Region	−13.552** (6.297)	−14.691** (6.200)	−9.491 (5.877)	−11.354** (5.527)		
=1 if Northern Region	−6.595 (5.849)	−7.771 (5.885)	−3.500 (5.197)	−6.016 (5.051)		
Season dummies?	Yes	Yes	No	No	Yes	Yes
HH fixed effects?	No	No	No	No	Yes	Yes
Constant	26.672*** (5.757)	20.237** (8.560)	33.488*** (4.560)	33.176*** (7.026)	34.199*** (1.223)	39.885*** (8.604)
Observations	2247	2247	4481	4481	4481	4481
R^2^	0.035	0.051	0.026	0.048	0.030	0.035
Number of households					1245	1245

Notes: For simplicity, the first row shows the ITT estimates for all estimators with and without covariates. Columns (1) and (2) are the simple mean difference estimates ($\tau_{SMD}$); columns (3) and (4) are the DID treatment effect estimates $\left(\tau_{DID}\right)$, which is the ‘treated*post-intervention’ interaction term; and columns (5) and (6) are the treatment effects estimates from the FE estimator $\left(\tau_{FE}\right)$. Robust standard errors, clustered at the LC1 level, are shown in parentheses. ***p < 0.01, **p < 0.05, *p < 0.1.

Overall, our findings appear to support the rationality of smallholder behavior on the adoption or dis-adoption of high-yielding varieties, in that if smallholders can effectively store maize grain from improved maize varieties, they will be more likely to plant these varieties and allocate a larger share of land to these varieties in the future. These results are similar in magnitude to previous findings in Ricker-Gilbert and Jones (2015) from Malawi, despite the fact that we use an experimental design with data from a different country in SSA. Indeed, it seems that storability concerns may be inhibiting the cultivation and diffusion of the higher-yielding maize varieties being promoted in SSA, as evidenced in our study.

### 6.4 Fertilizer use

Table 5 presents the ITT effects of a randomly offered 100 kg hermetic storage bags on the use of inorganic fertilizer. Considering that we found, on average, a higher likelihood of cultivating higher-yielding varieties among treated households, we would expect these varieties to be cultivated using inorganic fertilizer as this would be optimal for increasing yields. Although results from the three estimators are positive, they are small in magnitude and not statistically significant. Our results suggest that there is no direct link between improved storage technologies and inorganic fertilizer use. This makes sense as the improved storage bags offered to participants in our study makes it safer to plant and store hybrid maize, but has no direct benefit to fertilizer use, even if hybrid seeds and inorganic fertilizer have a positive and complementary agronomic relationship to yields. In fact, fertilizer use in Uganda is generally low and it is not uncommon to find households cultivating modern seed varieties without using fertilizer (Matsumoto and Yamano, 2011; Sheahan and Barrett, 2014).

**Table 5:** Treatment effects on households using inorganic fertilizer.

Dependent variable:=1 if HH used inorganic fertilizer	(1)	(2)	(3)	(4)	(5)	(6)
	SMD	SMD	DiD	DiD	FE	FE
Treatment effect (τ)	0.013 (0.024)	0.013 (0.024)	0.009 (0.035)	0.011 (0.035)	0.025 (0.037)	0.026 (0.036)
Household size		0.004 (0.004)		0.006 (0.004)		0.001 (0.003)
Age of household head		−0.002*** (0.001)		−0.002*** (0.001)		0.000 (0.000)
=1 if HH head is educated		−0.026 (0.050)		0.006 (0.028)		−0.036 (0.031)
Female headed household		−0.057** (0.022)		−0.046*** (0.015)		0.019 (0.032)
=1 if Eastern Region	−0.101** (0.050)	−0.111** (0.046)	−0.068 (0.042)	−0.080* (0.042)		
=1 if Western Region	−0.231*** (0.032)	−0.252*** (0.034)	−0.194*** (0.032)	−0.212*** (0.034)		
=1 if Northern Region	−0.216*** (0.033)	−0.238*** (0.035)	−0.190*** (0.032)	−0.210*** (0.035)		
Season dummies?	Yes	Yes	No	No	Yes	Yes
HH fixed effects?	No	No	No	No	Yes	Yes
Constant	0.258*** (0.032)	0.393*** (0.076)	0.206*** (0.032)	0.268*** (0.060)	0.117*** (0.008)	0.132*** (0.043)
Observations	2247	2247	4478	4478	4478	4478
R^2^	0.081	0.103	0.072	0.090	0.009	0.010
Number of households					1245	1245

Notes: For simplicity, the first row shows the ITT estimates for all estimators with and without covariates. Columns (1) and (2) are the simple mean difference estimates ($\tau_{SMD}$); columns (3) and (4) are the DID treatment effect estimates $\left(\tau_{DID}\right)$, which is the ‘treated*post-intervention’ interaction term; and columns (5) and (6) are the treatment effects estimates from the FE estimator $\left(\tau_{FE}\right)$. Robust standard errors, clustered at the LC1 level, are shown in parentheses. ***p < 0.01, **p < 0.05, *p < 0.1.

### 6.5 LATE effects on main outcomes (take-up)

Table 6 presents the LATE estimates across the three main outcomes tested when receipt of the hermetic bag is used as an IV for using the bag to store maize.^14^ In columns (1) and (2), we present LATE estimates on the decision to cultivate improved seeds without and with covariates, respectively. With both estimates at 13.6 percentage point increase in the likelihood of cultivating higher-yielding maize varieties, the LATE effects are higher than the ITT effects estimate at 9.7 percentage point (see Table 3). On share of area cultivated to the improved seed varieties in columns (3) and (4), we find a similar result to the binary decision to plant improved seed varieties. The LATE estimates, at 13.4 percentage point increase in share of area cultivated to improved varieties, are higher than the ITT at 9.5 percentage points (see Table 4). Lastly, on inorganic fertilizer use, although the LATE estimates in columns (5) and (6) are higher in magnitude than ITT estimates (Table 5), they are also not statistically significant.

**Table 6:** Local average treatment effects on three main outcomes.

Main outcome variables:	(1)	(2)	(3)	(4)	(5)	(6)
	Improved Seed	Improved Seed	Acreage Share	Acreage Share	Fertilizer Use	Fertilizer Use
LATE effects	0.136** (0.059)	0.134** (0.059)	13.337** (5.580)	13.169** (5.646)	0.018 (0.034)	0.018 (0.033)
Household size		0.011*** (0.004)		1.131*** (0.388)		0.004 (0.004)
Age of household head		−0.000 (0.001)		−0.028 (0.071)		−0.002*** (0.001)
=1 if HH head is educated		0.038 (0.056)		2.993 (5.596)		−0.026 (0.050)
Female headed household		−0.065** (0.031)		−6.573** (3.074)		−0.058*** (0.020)
=1 if Eastern Region	0.033 (0.077)	0.020 (0.077)	3.473 (7.483)	2.216 (7.478)	−0.101** (0.050)	−0.111** (0.046)
=1 if Western Region	−0.141** (0.064)	−0.152** (0.063)	−13.475** (6.275)	−14.564** (6.163)	−0.231*** (0.032)	−0.252*** (0.033)
=1 if Northern Region	−0.075 (0.060)	−0.087 (0.060)	−6.370 (5.870)	−7.506 (5.884)	−0.216*** (0.033)	−0.237*** (0.035)
Constant	0.277*** (0.059)	0.208** (0.087)	26.598*** (5.722)	19.869** (8.471)	0.258*** (0.032)	0.393*** (0.075)
Observations	2247	2247	2247	2247	2247	2247
R-squared	0.028	0.044	0.028	0.043	0.082	0.103

Notes: For each main outcome, the first columns show parsimonious estimates, whereas the second columns show estimates with additional covariates. Robust standard errors, clustered at the LC1 level, are shown in parentheses. ***p < 0.01, **p < 0.05, *p < 0.1.

### 6.6 Results for intermediate outcomes

To investigate some possible causal channels through which access to an improved storage technology may influence the cultivation of higher-yielding maize varieties as highlighted above, we examine a number of storage practices and self-reported storage losses below.

### 6.7 Quantity of maize stored

Table 7 presents the ITT effect of a randomly offered 100 kg hermetic storage bags on the quantity of maize stored at harvest. Without any covariate in the estimated ITT, column (1) shows the treatment group stored 125 kg more maize at harvest. However, this effect is not statistically significant. Moreover, when we controlled for total maize quantity harvested (households could only store or use storage technologies if they harvested maize) along with other household characteristics in column (2), the additional quantity stored reduced from 125 to 40 kg, and it is not statistically significant either.

**Table 7:** Treatment effects on quantity of maize stored at harvest (kg).

Dependent variable: Quantity stored at harvest (kg)	(1)	(2)	(3)	(4)	(5)	(6)
	SMD	SMD	DiD	DiD	FE	FE
Treatment effect (τ)	124.965 (101.639)	40.184 (31.908)	10.050 (139.101)	−70.084 (69.896)	1.521 (57.362)	−19.583 (46.450)
Total quantity harvested (kg)		0.701*** (0.090)		0.705*** (0.073)		0.409*** (0.074)
Household size		−25.696** (12.329)		−8.493 (7.262)		−1.643 (12.223)
Age of household head		−0.026 (0.686)		0.952 (0.959)		−0.567 (0.943)
=1 if HH head is educated		156.672* (78.338)		106.467** (51.670)		64.000 (42.739)
Female headed household		−17.766 (40.130)		3.196 (33.879)		−25.471 (44.442)
=1 if Eastern Region	−343.960** (161.936)	79.264** (36.188)	−347.929*** (124.690)	44.489 (57.043)		
=1 if Western Region	40.637 (187.661)	48.337 (47.004)	44.121 (171.781)	88.066* (46.256)		
=1 if Northern Region	−168.404 (169.215)	83.084** (33.855)	−170.719 (131.474)	92.333** (37.070)		
Season dummies?	Yes	Yes	No	No	Yes	Yes
HH fixed effects?	No	No	No	No	Yes	Yes
Constant	594.520*** (157.655)	−48.592 (103.918)	739.846*** (120.669)	−155.555 (135.019)	600.890*** (21.085)	226.424** (105.582)
Observations	2088	2080	4452	4310	4407	4265
R^2^	0.031	0.681	0.028	0.690	0.031	0.336
Number of households					1241	1240

Notes: For simplicity, the first row shows the ITT estimates for all estimators with and without covariates. Columns (1) and (2) are the simple mean difference estimates ($\tau_{SMD}$); columns (3) and (4) are the DID treatment effect estimates $\left(\tau_{DID}\right)$, which is the ‘treated*post-intervention’ interaction term; and columns (5) and (6) are the treatment effects estimates from the FE estimator $\left(\tau_{FE}\right)$. Robust standard errors, clustered at the LC1 level, are shown in parentheses. ***p < 0.01, **p < 0.05, *p < 0.1.

The ITT estimates from our preferred DiD and FE estimators in columns (3–6) show there is no statistically significant impact of the treatment on maize quantity stored at harvest. While this result may seem surprising given the nature of our intervention, we believe that there are several behavioral reasons to explain it. First, because treated households received only one 100 kg capacity hermetic bag, and the average household stored 606 kg of maize at baseline, the intervention did relatively little to increase total storage capacity of the household. In fact, Table B.3 in the appendix suggests that receipt of one hermetic bag has no statistically significant impact on storage capacity. Second, since maize is the staple crop for most households and the bag did not significantly increase total storage capacity, treated households likely adopted a safety-first approach where they stored the amount of maize necessary for their consumption in the improved hermetic storage bag, instead of in a less effective traditional bag.

Third, subsequent results show that, on average, treated households significantly increased their length of storage for consumption by 21 percent, and reported about 75 percent reduction in storage losses. These findings further support the safety-first utility approach that households were primarily concerned with consumption.^15^ Fourth, storage is highly correlated with production. Given that households reported a significant (30 percent) drop in production from baseline to follow-up survey, due to drought in the seasons following our intervention, quantity stored also decreased across treatment groups in general. Regardless, we may ultimately be underpowered to pick up statistical significance of the treatment intervention on quantity stored. Indeed, the standard deviation on this variable far exceeds the mean. Moreover, few studies are powered enough to pick up this type of effect as pointed out in a recent article in Kenya by Aggarwal et al. (2017).

Lastly, smallholders decide to store grains under multiple binding constraints. These include efficient storage technology and liquidity constraints. By providing access to an improved storage technology, we solved a part of the constraint but not all. Thus, even among treated households, liquidity constraint at harvest could have played a role in the lack of increase in quantity stored whereby treated households only stored for consumption, but sell the remaining grains to meet immediate liquidity needs (Stephens and Barrett, 2011).

### 6.8 Length of storage for consumption and sales

Table 8 presents the ITT effects on the length of storage if a household stores maize at harvest with the intention of using it for its own consumption. With the SMD estimator in column (1), on average, households who were randomly treated with an improved storage technology store for about 1.6 weeks longer than the control group of households (significant at p-value<0.05). This effect remains consistent when we added covariates in column (2).

**Table 8:** Treatment effects on length of storage for consumption purpose (weeks).

Dependent variable: Length of storage for consumption (weeks)	(1)	(2)	(3)	(4)	(5)	(6)
	SMD	SMD	DiD	DiD	FE	FE
Treatment effect (τ)	1.548** (0.752)	1.558** (0.755)	3.003*** (0.759)	3.006*** (0.770)	2.977*** (0.775)	2.963*** (0.779)
Household size		0.172* (0.086)		0.115** (0.055)		0.038 (0.086)
Age of household head		−0.030** (0.014)		−0.033*** (0.009)		−0.023 (0.021)
=1 if HH head is educated		0.026 (0.864)		0.882 (0.537)		−0.394 (1.168)
Female headed household		−0.192 (0.684)		0.183 (0.457)		1.641 (1.237)
=1 if Eastern Region	2.545*** (0.943)	2.298** (0.956)	2.398*** (0.882)	2.228** (0.911)		
=1 if Western Region	2.488** (0.984)	2.209** (0.910)	1.542 (0.932)	1.310 (0.909)		
=1 if Northern Region	1.962* (0.987)	1.664* (0.960)	−0.862 (0.797)	−1.172 (0.807)		
Season dummies?	Yes	Yes	No	No	Yes	Yes
HH fixed effects?	No	No	No	No	Yes	Yes
Constant	13.495*** (0.635)	13.959*** (1.140)	13.781*** (0.665)	13.917*** (0.962)	15.100*** (0.261)	15.934*** (1.457)
Observations	2088	2088	4451	4451	4451	4451
R^2^	0.022	0.030	0.027	0.039	0.023	0.024
Number of households					1244	1244

Notes: For simplicity, the first row shows the ITT estimates for all estimators with and without covariates. Columns (1) and (2) are the simple mean difference estimates ($\tau_{SMD}$); columns (3) and (4) are the DID treatment effect estimates $\left(\tau_{DID}\right)$, which is the ‘treated*post-intervention’ interaction term; and columns (5) and (6) are the treatment effects estimates from the FE estimator $\left(\tau_{FE}\right)$. Robust standard errors, clustered at the LC1 level, are shown in parentheses. ***p < 0.01, **p < 0.05, *p < 0.1.

In columns (3) and (4), without and with covariates respectively, the ITT estimates from the DiD estimator is, on average, three additional weeks of storage for consumption among treated households. Likewise, the FE estimates are virtually the same as the DiD estimates. Thus, we find evidence that a random offer of 100 kg capacity hermetic bag extends a household's length of storage for consumption. Because the average length of storage for consumption purposes was 14 weeks at baseline, this effect implies a 21 percent increase in storage period for consumption, which could have a significant impact on a household's ability to feed itself. Our findings are consistent with previous literature such as (Gitonga et al., 2013) where hermetic metal silo adopters were able to store their maize for 1.8–2.4 months longer in Kenya.^16^

In addition, given that the reported average length of the lean period or duration of food scarcity among sampled households is about eight weeks, the ITT effect of a single 100 kg improved storage technology, which is three additional weeks of storage for consumption, could reduce households' lean period by as much as 38%—a potentially meaningful impact.

Table 9 presents the ITT effects of receiving an improved storage technology on length of time households store maize with the aim of selling later in the post-harvest period. ITT estimate suggests that receiving one hermetic storage bag causes households to store 0.6–0.7 weeks longer for sale on average. Although the SMD estimates in columns (1) and (2) are not statistically significant, they are similar to estimates from the other estimators in columns (3) through (6), where the estimates are marginally significant at p-value<0.10. Given that storage for sales is about 4 weeks, a marginal increase of 0.7 week is about 17–18 percent of the average storage period for sale.

**Table 9:** Treatment effects on length of storage for sale purpose (weeks).

Dependent variable: Length of storage for sales (weeks)	(1)	(2)	(3)	(4)	(5)	(6)
	SMD	SMD	DiD	DiD	FE	FE
Treatment effect (τ)	0.413 (0.316)	0.345 (0.310)	0.620* (0.373)	0.582 (0.381)	0.692* (0.354)	0.645* (0.366)
Household size		−0.049 (0.044)		0.010 (0.042)		−0.129 (0.079)
Age of household head		−0.010 (0.010)		−0.022** (0.009)		0.011 (0.012)
=1 if HH head is educated		0.771** (0.324)		1.029*** (0.271)		−0.019 (0.610)
Female headed household		−1.056*** (0.328)		−0.085 (0.295)		0.639 (0.480)
=1 if Eastern Region	−0.674** (0.310)	−0.643** (0.315)	−0.307 (0.354)	−0.337 (0.343)		
=1 if Western Region	0.952** (0.396)	0.821** (0.393)	1.652*** (0.466)	1.503*** (0.436)		
=1 if Northern Region	2.231*** (0.398)	2.082*** (0.394)	2.154*** (0.362)	1.940*** (0.344)		
Season dummies?	Yes	Yes	No	No	Yes	Yes
HH fixed effects?	No	No	No	No	Yes	Yes
Constant	2.737*** (0.246)	3.095*** (0.675)	3.608*** (0.318)	3.768*** (0.624)	4.682*** (0.194)	4.928*** (0.975)
Observations	2088	2088	4450	4450	4450	4450
R^2^	0.040	0.053	0.045	0.060	0.030	0.033
Number of households					1244	1244

Notes: For simplicity, the first row shows the ITT estimates for all estimators with and without covariates. Columns (1) and (2) are the simple mean difference estimates ($\tau_{SMD}$); columns (3) and (4) are the DID treatment effect estimates $\left(\tau_{DID}\right)$, which is the ‘treated*post-intervention’ interaction term; and columns (5) and (6) are the treatment effects estimates from the FE estimator $\left(\tau_{FE}\right)$. Robust standard errors, clustered at the LC1 level, are shown in parentheses. ***p < 0.01, **p < 0.05, *p < 0.1.

Although the ITT effect on the duration of storage for sale is nuanced, the impact on the duration of maize storage for consumption is more pronounced. Therefore, we find evidence to support the longer storage period causal pathway through which improved storage technology causes households to plant more improved maize varieties, particularly for consumption purposes.

### 6.9 Storage chemical use

One of the major benefits of using hermetic storage technology is that it alleviates the need to apply chemical insecticides on stored maize. Chemical insecticides can control storage insects, but could also be harmful to human health if used improperly (Golob, 2002; Williamson et al., 2008). In Table 10, we present ITT effects of being treated with a 100 kg capacity hermetic storage bag on this indicator. The SMD estimator in column (1) shows that treated households are, on average, less likely to apply storage chemicals by roughly 6.2 percentage points. When we include household covariates in column (2), the likelihood of treated households using less chemical insecticides did not change, indicating that the estimates are consistent. Both are statistically significant with a p-value <0.001. However, because of the ex-ante (statistically weak) imbalance of 3 percentage points between the treatment and control groups in the baseline (see Table 2), we are cautious about the estimates from the simple mean difference. Fortunately, the DiD and FE estimates shown in columns (3–4) and (5–6) respectively should remove the ex-ante bias between the two groups.

**Table 10:** Treatment effects on if households use storage chemicals.

Dependent variable:=1 if HH use storage chemical on maize	(1)	(2)	(3)	(4)	(5)	(6)
	SMD	SMD	DiD	DiD	FE	FE
Treatment effect (τ)	−0.062*** (0.016)	−0.064*** (0.017)	−0.041*** (0.013)	−0.041*** (0.013)	−0.036** (0.015)	−0.037** (0.016)
Household size		0.003 (0.002)		−0.001 (0.002)		−0.002 (0.005)
Age of household head		0.000 (0.001)		−0.001 (0.001)		0.000 (0.001)
=1 if HH head is educated		0.037 (0.025)		0.034* (0.017)		−0.012 (0.046)
Female headed household		−0.025** (0.012)		−0.011 (0.016)		0.042 (0.051)
=1 if Eastern Region	0.042*** (0.011)	0.040*** (0.011)	0.044*** (0.014)	0.045*** (0.014)		
=1 if Western Region	0.095*** (0.030)	0.093*** (0.030)	0.057** (0.028)	0.053* (0.028)		
=1 if Northern Region	0.036** (0.018)	0.032* (0.018)	0.041*** (0.013)	0.034** (0.014)		
Season dummies?	Yes	Yes	Yes	Yes	Yes	Yes
HH fixed effects?	No	No	No	No	Yes	Yes
Constant	0.044*** (0.011)	−0.004 (0.050)	0.072*** (0.014)	0.087** (0.040)	0.093*** (0.001)	0.093 (0.067)
Observations	2371	2371	4733	4733	4733	4733
R^2^	0.026	0.032	0.012	0.017	0.002	0.003
Number of households					1245	1245

Notes: For simplicity, the first row shows the ITT estimates for all estimators with and without covariates. Columns (1) and (2) are the simple mean difference estimates ($\tau_{SMD}$); columns (3) and (4) are the DID treatment effect estimates $\left(\tau_{DID}\right)$, which is the ‘treated*post-intervention’ interaction term; and columns (5) and (6) are the treatment effects estimates from the FE estimator $\left(\tau_{FE}\right)$. Robust standard errors, clustered at the LC1 level, are shown in parentheses. ***p < 0.01, **p < 0.05, *p < 0.1.

Both estimators suggest that the ex-ante bias between groups has been removed. The results show that the treated households are still less likely to use storage chemicals by about 4 percentage points on average. The estimates with and without covariates are similar in both estimators indicating consistency, and are significant at p-value<0.05. Thus, we are confident that households consider hermetic bags as an alternative to chemical insecticides, at least partly. This impact suggests positive health benefits from using a chemical-free improved storage technology because of the potential hazards associated with using chemical insecticides on food that will be consumed. Anecdotal evidence suggest that households may be applying unregulated chemical insecticides on maize, and even when they are regulated, households may consume their maize before the chemical's latency period has elapsed.

### 6.10 Storage losses

Table 11 shows how access to an improved storage technology affects self-reported on-farm storage losses at the technology level.^17^ Since treated households were offered a 100 kg hermetic storage bag, households likely stored a larger proportion of their harvested maize in other non-hermetic technologies. Thus, it is important to examine storage losses in the hermetic bags compared to storage losses in other technologies within the households. In column (1), the SMD shows that on average, self-reported storage loss is 2.2 percentage points less among treated households— about 61% of the average post-harvest storage loss reported in our sample. The result is the same when we added covariates in column (2), indicating consistency of the estimates. Both are also statistically significant at p-value<0.001.

**Table 11:** Treatment effects on on-farm storage losses.

Dependent variable: Self-reported on-farm storage losses (%)	(1)	(2)	(3)	(4)	(5)	(6)
	SMD	SMD	DiD	DiD	FE	FE
Treatment effect (τ)	−2.200*** (0.570)	−2.190*** (0.581)	−2.325** (0.982)	−2.223** (0.992)	−2.297* (1.267)	−2.432* (1.223)
Household size		0.060 (0.044)		0.096 (0.063)		0.005 (0.119)
Age of household head		−0.015* (0.008)		−0.017* (0.010)		−0.003 (0.016)
=1 if HH head is educated		0.078 (0.537)		−0.014 (0.344)		2.301* (1.222)
Female headed household		0.141 (0.518)		−0.446 (0.364)		2.352*** (0.793)
=1 if Eastern Region	0.707 (0.584)	0.612 (0.580)	0.322 (0.626)	0.146 (0.644)		
=1 if Western Region	−0.015 (0.510)	−0.134 (0.504)	−0.345 (0.655)	−0.529 (0.656)		
=1 if Northern Region	−0.207 (0.552)	−0.340 (0.527)	−0.708 (0.600)	−0.913 (0.612)		
Season dummies?	Yes	Yes	Yes	Yes	Yes	Yes
HH fixed effects?	No	No	No	No	Yes	Yes
Constant	3.005*** (0.437)	3.307*** (0.915)	3.931*** (0.715)	4.304*** (0.940)	3.471*** (0.205)	1.208 (0.916)
Observations	2086	2086	4217	4217	4217	4217
R^2^	0.011	0.014	0.008	0.012	0.009	0.014
Number of households					1242	1242

Notes: For simplicity, the first row shows the ITT estimates for all estimators with and without covariates. Columns (1) and (2) are the simple mean difference estimates ($\tau_{SMD}$); columns (3) and (4) are the DID treatment effect estimates $\left(\tau_{DID}\right)$, which is the ‘treated*post-intervention’ interaction term; and columns (5) and (6) are the treatment effects estimates from the FE estimator $\left(\tau_{FE}\right)$. Robust standard errors, clustered at the LC1 level, are shown in parentheses. ***p < 0.01, **p < 0.05, *p < 0.1.

In columns (3) and (4), the DiD estimated effects are not different from the SMD reported in columns (1) and (2) but the precision decreased as both estimates are now significant at p-value<0.05. In columns (5), the FE estimate without covariates shows that on-farm storage loss is reduced by 2.3 percentage points for maize stored in the technology within treated households relative to other storage technologies. Likewise, in column (6) with the addition of covariates, the magnitude is about the same as in column (5). Both estimates are statistically significant at p-value<0.1. Thus, these estimates suggest that about 70% of the average reported losses are eliminated within hermetic storage bags for treated households. These results are supported by previous findings in (Bokusheva et al., 2012; Gitonga et al., 2013) who find that the major effect of hermetic metal silos for users is the near complete elimination of losses due to storage insect pest attacks.

## 7 Robustness checks

We conducted a number of robustness checks to ensure validity of our results. First, we re-estimated our treatment effects for the main outcome variables with Lee bounds to account for potential attrition bias. The point estimates from our results statistically fall within the estimated Lee bounds, indicating that the estimated effects are not affected by attrition bias (see Appendix Table A.2 for Lee bounds estimates).

Second, we conducted multiple-hypothesis correction testing for all outcome variables. Appendix Table A.4 presents the sharpened q-values as implemented by Anderson (2008) and the unadjusted p-values from the Huber-White robust standard errors, clustered at the LC1 levels. Our conclusion from the multiple-hypothesis correction is that our findings are robust to the corrections. For example, the q-values for the decision to cultivate higher-yielding varieties as well as share of area planted to these varieties remain significant at 5 percent test levels.

Third, one potential challenge to the validity of our results is if there is a contamination of our experimental design. Members of the control group could have purchased the bags, while members of the treatment group could have purchased additional bags beyond the one they were given as part of the experiment. The former type of contamination could lead to attenuation bias, while the latter would lead to an over estimation of impacts. In fact, about 11 percent of the treated households reported buying one or more additional bag with their own money, whereas only 6.4 percent of the control group bought one or more bags with their own money. The supply chain for the improved hermetic bags remained limited between our intervention in 2015 and follow-up survey in 2016, so purchasing of the bags outside of our intervention was low. Regardless, we test the main results (cultivation of improved maize seed and share of area planted) for consistency in terms of magnitude and statistical significance across specifications, by dropping contaminated households (who bought bag(s) outside our intervention) in the treatment and control group.

Results of these other robustness checks are presented in Appendix B. Overall, our results are consistent when contaminated households were dropped from the analysis. Excluding contaminated observations from both groups in our estimation does not change the coefficient estimates in the DiD and FE estimators (Table B.1a, Table B.2a). These estimators use the baseline and post-intervention data to deal with unobserved factors that may affect contamination and the decision to plant improved maize varieties. In addition, the results are largely consistent for the SMD estimator. Furthermore, including observations from the control group in our estimation attenuated our full sample estimate (Table B.2b, Table B.1b). The converse is the case when we included contaminated observations from the treatment group, as ITT effects from the full-sample estimates are slightly bigger than estimates without contaminated observations from the treatment group (Table B.2c, Table B.1c). In general, these results, which are largely consistent across the estimators, speak to the additional robustness benefits that having a baseline and post-intervention data provide. They further re-affirm our confidence in the treatment effects as estimated using the full sample above.

## 8 Conclusions and policy recommendations

We used a randomized controlled trial to estimate the impacts of an improved storage technology—a hermetic (airtight) bag—on smallholder farm households in Uganda. To our knowledge, this is one of the first large-scale RCTs to evaluate a post-harvest storage technology and the first to evaluate how storage technology affects smallholders' input use and planting decisions in the developing world. The main behavioral hypothesis tested is whether the improved storage technology is causally linked to production decisions and storage practices in the subsequent year. Our results indicate that receiving one hermetic bag that can store 100 kg of shelled maize has a direct and positive impact on households' decisions to cultivate improved maize varieties that are higher-yielding but more susceptible to insect pests in storage, than traditional maize varieties.

Our results further indicate that treated households did not store more maize relative to the control group. We suspect this is mainly because households generally adopted a safety-first approach, storing maize—a major staple food—for consumption in an improved technology; and those households with liquidity constraints may choose to sell their remaining maize at harvest rather than store in inefficient traditional storage technologies. However, the treated households stored maize for longer periods for both consumption and marketed sales. We conclude that improved storage technology has the ability to reduce food insecurity by increasing the duration of storage for consumption. The treated household in our RCT who received one hermetic bag, stored 20 percent longer on average, and results could be larger if they purchase additional hermetic bags in the future. Furthermore, we find evidence that the treated households are less likely to use chemical insecticides on their stored maize, avoiding the potential health hazards typically associated with improper use of these chemicals on their food supply. Lastly, the intervention also reduced average self-reported storage losses by 61–70 percent, increasing household food supply and availability.

The main policy recommendation that emanates from our study is that development agencies, researchers and policy makers advocating the use of improved higher-yielding hybrid maize seeds among smallholder farmers in SSA should consider promoting hermetic storage technologies as a complementary intervention. The use of improved storage technologies can help allay smallholders' concerns about these softer-kernel hybrid maize varieties, which are susceptible to pest attacks in storage. This may be the missing link needed to convince rational farmers to take-up these higher-yielding varieties. Our experimental results show that offering a household one free hermetic bag that holds a maximum of 100 kg can have a meaningful impact on household wellbeing in terms of duration of storage, reduction in use of storage chemicals and storage losses, which translates into the adoption of productivity enhancing seeds in the future. The impacts would likely be even larger for households acquiring multiple bags, who could then store a substantially larger share of their harvest in an insect-free and chemical-free environment. Though we have identified some important shorter-term benefits from hermetic technology, future work should consider and estimate how the use of these bags affects income, consumption, nutrition, and dietary diversity over a longer period.

## Figures and Tables

**Table A.1:** Mean difference at baseline between attritted and returning households.

Variables	Returning		Attritted		
	Mean	SD	Coeff.	p-value	N
	(1)	(2)	(3)	(4)	(5)
*Dependent Variables*					
Quantity stored (kg)	609	1071	108	0.575	2364
Length of storage for consumption (weeks)	14.26	9.58	−0.643	0.655	2364
Length of storage for sales (weeks)	4.38	5.99	−0.332	0.552	2364
Reported actual post-harvest losses (%)	3.21	6.60	0.741	0.242	2131
=1 if HH used storage chemical on maize	0.11	0.31	0.071	0.132	2362
=1 if HH planted improved maize seed	0.34	0.47	−0.006	0.923	2235
=1 if HH used inorganic fertilizer	0.09	0.29	0.003	0.932	2231
Share of improved maize area (%)	33.27	46.93	−1.152	0.839	2234
*Household Characteristics*					
Age of household head (years)	45.0	14.7	−5.96***	0.001	2380
Household size	6.4	3.1	−0.67*	0.099	2380
=1 if female-headed household	0.16	0.37	0.02	0.631	2380
=1 if Polygamous	0.17	0.38	−0.01	0.835	2380
=1 if HH head has any form of education	0.88	0.32	0.02	0.512	2380
=1 if HH has radio	0.78	0.41	−0.07	0.230	2370
=1 if HH has mobile phone	0.69	0.46	−0.01	0.851	2370
*Production and PH practices*					
Total maize area (ha.)	0.52	0.48	0.11	0.236	2235
Total quantity harvested-maize (kg)	905	1259	364	0.160	2235
*Region Effects*					
=1 if REGION = 200, Eastern	0.25	0.43	0.08	0.382	2380
=1 if REGION = 300, Northern	0.25	0.43	0.00	0.952	2380
=1 if REGION = 400, Western	0.25	0.43	−0.07	0.152	2380
=1 if REGION = 100, Central wo Kampala	0.25	0.43	−0.01	0.823	2380

Notes: Columns 1 and 2 report means and standard deviations for control villages in the baseline. Columns 3 through 5 report results from an OLS regression comparing households in treated and control villages in the baseline controlling for region effects and clustering standard errors at the village level. Columns 3 and 4 report the OLS coefficient and p-value corresponding to the binary treatment indicator and column 5 reports the sample size for each regression.

***p < 0.01, **p < 0.05, *p < 0.1.

**Table A.2:** Lee bounds estimates to account for attrition bias on treatment effects.

Main Outcome Variables	Coef.	Std. Err.
=1 if HH planted improved maize varieties		
lower	0.081***	0.025
upper	0.092***	0.024
Share of improved maize area (%)		
lower	7.903***	2.496
upper	9.012***	2.406
=1 if HH used inorganic fertilizer		
lower	−0.011	0.020
upper	0.000	0.018

**Table A.3:** Intra-cluster correlation coefficients for dependent variables.

variables	ICC	SE	N
Quantity Stored (kg)	0.013	0.008	4452
Length of storage for consumption (weeks)	0.045	0.019	4452
Length of storage for sales (weeks)	0.024	0.017	4450
Reported actual post-harvest losses (%)	0.000	0.010	4219
=1 if HH used storage chemicals	0.027	0.038	4733
=1 if HH planted improved maize seed	0.099	0.126	4482
Share of improved maize area (%)	0.103	0.127	4481
Total area planted to improved maize (ha.)	0.102	0.070	4482
=1 if HH uses inorganic fertilizer	0.014	0.021	4478

Notes: An ICC value of zero means there is no difference between the variation within clusters (LC1s) and the variation between clusters. The closer the ICC is to 1, the more observations (25 households) within each LC1 lack variation (implying no power gain or efficiency from having a larger sample). However, an ICC value closer to zero indicates a bigger variation in the within sample, which is beneficial in terms of efficiency or power gain.

Source: Authors' compilation.

**Table A.4:** Comparison of p-values and sharpened q-values for multiple hypothesis testing.

Outcome Variables	SMD		DID		FE	
	p-val	q-val	p-val	q-val	p-val	q-val
=1 if households planted improved maize varieties	0.027	0.043	0.046	0.059	0.031	0.058
Share of improved maize area (%)	0.022	0.043	0.033	0.053	0.022	0.055
=1 if households used inorganic fertilizer	0.598	0.290	0.797	0.295	0.513	0.172
Quantity of maize stored (kg)	0.225	0.107	0.943	0.309	0.979	0.325
Length of storage for consumption (weeks)	0.047	0.058	0.000	0.001	0.000	0.001
Length of storage for sales (kg)	0.199	0.107	0.103	0.074	0.057	0.063
=1 if households used storage chemicals	0.000	0.001	0.003	0.011	0.021	0.055
Self-reported storage losses (%)	0.000	0.001	0.022	0.047	0.053	0.063

Note: The sharpened q-values were computed from the unadjusted p-values derived from Huber-White robust standard errors, clustered at the LC1 level, following Anderson (2008).

**Table A.5:** Treatment effects on on-farm storage losses at the household level.

Dependent variable: Self-reported on-farm storage losses (%)	(1)	(2)	(3)	(4)	(5)	(6)
	SMD	SMD	DiD	DiD	FE	FE
Treatment effect (τ)	−0.017 (0.505)	−0.008 (0.510)	−0.300 (0.831)	−0.260 (0.864)	−0.287 (0.867)	−0.361 (0.844)
Household size		0.096** (0.039)		0.088 (0.057)		0.057 (0.123)
Age of household head		−0.019** (0.009)		−0.017* (0.009)		−0.005 (0.016)
=1 if HH head is educated		−0.023 (0.534)		−0.118 (0.333)		2.230* (1.311)
Female headed household		−0.021 (0.568)		−0.556 (0.353)		1.936*** (0.707)
=1 if Eastern Region	1.035* (0.593)	0.893 (0.584)	0.689 (0.701)	0.522 (0.716)		
=1 if Western Region	−0.007 (0.518)	−0.171 (0.510)	−0.287 (0.676)	−0.472 (0.693)		
=1 if Northern Region	−0.014 (0.553)	−0.191 (0.525)	−0.390 (0.670)	−0.590 (0.685)		
Season dummies?	No	Yes	Yes	Yes	Yes	Yes
HH fixed effects?	No	No	No	No	Yes	Yes
Constant	2.987*** (0.442)	3.381*** (0.936)	3.509*** (0.773)	4.036*** (0.980)	3.951*** (0.242)	1.560 (1.009)
Observations	2088	2088	4219	4219	4219	4219
R-squared	0.006	0.011	0.005	0.010	0.004	0.009
Number of HHs					1242	1242

Notes: For simplicity, the first row shows the ITT estimates for all estimators with and without covariates. Columns (1) and (2) are the simple mean difference estimates ($\tau_{SMD}$); columns (3) and (4) are the DID treatment effect estimates $\left(\tau_{DID}\right)$, which is the ‘treated*post-intervention’ interaction term; and columns (5) and (6) are the treatment effects estimates from the FE estimator $\left(\tau_{FE}\right)$. Robust standard errors, clustered at the LC1 level, are shown in parentheses. ***p < 0.01, **p < 0.05, *p < 0.1.

**Table B.1a:** Treatment effects on household planting improved maize varieties (sample without the treatment and control groups contaminated observations).

Dependent variable:=1 if HH planted improved maize	(1)	(2)	(3)	(4)	(5)	(6)
	SMD	SMD	DiD	DiD	FE	FE
Treatment effect (τ)	0.079** (0.036)	0.079** (0.036)	0.085* (0.049)	0.088* (0.049)	0.103** (0.040)	0.096** (0.041)
Household size		0.011*** (0.004)		0.006** (0.002)		−0.011** (0.005)
Age of household head		−0.000 (0.001)		−0.001 (0.001)		−0.001 (0.001)
=1 if HH head is educated		0.025 (0.058)		0.090** (0.034)		0.032 (0.036)
Female headed household		−0.072** (0.027)		−0.095*** (0.029)		0.120* (0.065)
=1 if Eastern Region	0.024 (0.075)	0.013 (0.076)	0.049 (0.060)	0.038 (0.057)	n/a	n/a
=1 if Western Region	−0.149** (0.061)	−0.160** (0.061)	−0.103* (0.058)	−0.121** (0.054)		
=1 if Northern Region	−0.074 (0.060)	−0.085 (0.060)	−0.040 (0.053)	−0.065 (0.052)		
Season dummies?	No	Yes	Yes	Yes	Yes	Yes
HH fixed-effects?	No	No	No	No	Yes	Yes
Constant	0.274*** (0.058)	0.215** (0.088)	0.342*** (0.046)	0.343*** (0.071)	0.347*** (0.012)	0.395*** (0.088)
Observations	2166	2166	4397	4397	4397	4397
R^2^	0.033	0.047	0.027	0.053	0.034	0.040
Number of households					1243	1243

Notes: For simplicity, the first row shows the ITT estimates for all estimators with and without covariates. Columns (1) and (2) are the simple mean difference estimates ($\tau_{SMD}$); columns (3) and (4) are the DID treatment effect estimates $\left(\tau_{DID}\right)$, which is the ‘treated*post-intervention’ interaction term; and columns (5) and (6) are the treatment effects estimates from the FE estimator $\left(\tau_{FE}\right)$. Robust standard errors, clustered at the LC1 level, are shown in parentheses. ***p < 0.01, **p < 0.05, *p < 0.1.

**Table B.1b:** Treatment effects on household planting improved maize varieties (sample without the control group contaminated observations).

Dependent variable:=1 if HH planted improved maize	(1)	(2)	(3)	(4)	(5)	(6)
	SMD	SMD	DiD	DiD	FE	FE
Treatment effect (τ)	0.103** (0.041)	0.102** (0.042)	0.104** (0.048)	0.106** (0.048)	0.107** (0.044)	0.101** (0.045)
Household size		0.011*** (0.004)		0.007*** (0.002)		−0.010* (0.005)
Age of household head		−0.000 (0.001)		−0.001 (0.001)		−0.001 (0.001)
=1 if HH head is educated		0.028 (0.058)		0.091*** (0.034)		0.032 (0.036)
Female headed household		−0.075*** (0.027)		−0.096*** (0.029)		0.119* (0.065)
=1 if Eastern Region	0.022 (0.076)	0.010 (0.076)	0.048 (0.060)	0.036 (0.057)	n/a	n/a
=1 if Western Region	−0.142** (0.064)	−0.153** (0.063)	−0.098 (0.059)	−0.117** (0.056)		
=1 if Northern Region	−0.079 (0.060)	−0.091 (0.060)	−0.044 (0.053)	−0.069 (0.052)		
Season dummies?	No	No	Yes	Yes	Yes	Yes
HH fixed-effects?	No	No	No	No	Yes	Yes
Constant	0.274*** (0.058)	0.207** (0.089)	0.342*** (0.046)	0.339*** (0.071)	0.349*** (0.012)	0.396*** (0.087)
Observations	2190	2190	4423	4423	4423	4423
R^2^	0.034	0.050	0.026	0.053	0.033	0.038
Number of households					1244	1244

Notes: For simplicity, the first row shows the ITT estimates for all estimators with and without covariates. Columns (1) and (2) are the simple mean difference estimates ($\tau_{SMD}$); columns (3) and (4) are the DID treatment effect estimates $\left(\tau_{DID}\right)$, which is the ‘treated*post-intervention’ interaction term; and columns (5) and (6) are the treatment effects estimates from the FE estimator $\left(\tau_{FE}\right)$. Robust standard errors, clustered at the LC1 level, are shown in parentheses. ***p < 0.01, **p < 0.05, *p < 0.1.

**Table B.1c:** Treatment effects on household planting improved maize varieties (sample without the treatment group contaminated observations).

Dependent variable:=1 if HH planted improved maize	(1)	(2)	(3)	(4)	(5)	(6)
	SMD	SMD	DiD	DiD	FE	FE
Treatment effect (τ)	0.072* (0.036)	0.072* (0.037)	0.079 (0.049)	0.082* (0.049)	0.095** (0.041)	0.088** (0.042)
Household size		0.011*** (0.004)		0.006*** (0.002)		−0.011** (0.005)
Age of household head		−0.000 (0.001)		−0.001* (0.001)		−0.000 (0.001)
=1 if HH head is educated		0.033 (0.057)		0.093*** (0.033)		0.028 (0.036)
Female headed household		−0.064** (0.030)		−0.091*** (0.030)		0.121* (0.066)
=1 if Eastern Region	0.034 (0.077)	0.022 (0.077)	0.054 (0.060)	0.043 (0.057)	n/a	n/a
=1 if Western Region	−0.148** (0.062)	−0.160** (0.061)	−0.101* (0.058)	−0.120** (0.054)		
=1 if Northern Region	−0.071 (0.059)	−0.084 (0.060)	−0.039 (0.053)	−0.065 (0.051)		
Season dummies?	No	No	Yes	Yes	Yes	Yes
HH fixed-effects?	No	No	No	No	Yes	Yes
Constant	0.278*** (0.058)	0.219** (0.087)	0.342*** (0.046)	0.342*** (0.071)	0.347*** (0.012)	0.398*** (0.089)
Observations	2223	2223	4456	4456	4456	4456
R^2^	0.035	0.056	0.026	0.053	0.030	0.036
Number of households					1244	1244

Notes: For simplicity, the first row shows the ITT estimates for all estimators with and without covariates. Columns (1) and (2) are the simple mean difference estimates ($\tau_{SMD}$); columns (3) and (4) are the DID treatment effect estimates $\left(\tau_{DID}\right)$, which is the ‘treated*post-intervention’ interaction term; and columns (5) and (6) are the treatment effects estimates from the FE estimator $\left(\tau_{FE}\right)$. Robust standard errors, clustered at the LC1 level, are shown in parentheses. ***p < 0.01, **p < 0.05, *p < 0.1.

**Table B.2a:** Treatment effects on share of area planted to improved varieties (sample without the treatment and control groups contaminated observations).

Dependent variable: Share of improved maize area (%)	(1)	(2)	(3)	(4)	(5)	(6)
	SMD	SMD	DiD	DiD	FE	FE
Treatment effect (τ)	8.154** (3.541)	8.181** (3.625)	9.089* (4.822)	9.402* (4.841)	10.936*** (3.978)	10.247** (4.056)
Household size		1.067*** (0.369)		0.609*** (0.224)		−1.132** (0.500)
Age of household head		−0.014 (0.073)		−0.129 (0.083)		−0.064 (0.118)
=1 if HH head is educated		1.738 (5.773)		8.356** (3.369)		3.228 (3.637)
Female headed household		−7.336*** (2.624)		−9.434*** (2.865)		11.356* (6.385)
=1 if Eastern Region	2.630 (7.239)	1.455 (7.335)	5.032 (5.850)	3.921 (5.585)	n/a	n/a
=1 if Western Region	−14.048** (6.031)	−15.079** (6.011)	−9.917* (5.734)	−11.730** (5.405)		
=1 if Northern Region	−6.268 (5.866)	−7.279 (5.949)	−3.307 (5.242)	−5.732 (5.117)		
Season dummies?	No	No	Yes	Yes	Yes	Yes
HH fixed-effects?	No	No	No	No	Yes	Yes
Constant	26.290*** (5.618)	20.452** (8.675)	33.571*** (4.485)	33.710*** (7.040)	34.098*** (1.161)	39.703*** (8.716)
Observations	2166	2166	4396	4396	4396	4396
R^2^	0.032	0.046	0.027	0.052	0.034	0.040
Number of households					1243	1243

Notes: For simplicity, the first row shows the ITT estimates for all estimators with and without covariates. Columns (1) and (2) are the simple mean difference estimates ($\tau_{SMD}$); columns (3) and (4) are the DID treatment effect estimates $\left(\tau_{DID}\right)$, which is the ‘treated*post-intervention’ interaction term; and columns (5) and (6) are the treatment effects estimates from the FE estimator $\left(\tau_{FE}\right)$. Robust standard errors, clustered at the LC1 level, are shown in parentheses. ***p < 0.01, **p < 0.05, *p < 0.1.

**Table B.2b:** Treatment effects on share of area planted to improved varieties (sample without the control group contaminated observations).

Dependent variable: Share of improved maize area (%)	(1)	(2)	(3)	(4)	(5)	(6)
	SMD	SMD	DiD	DiD	FE	FE
Treatment effect (τ)	10.171** (3.916)	10.073** (3.969)	10.798** (4.681)	10.980** (4.690)	11.104** (4.252)	10.535** (4.399)
Household size		1.135*** (0.402)		0.652*** (0.229)		−1.025* (0.532)
Age of household head		−0.012 (0.076)		−0.128 (0.084)		−0.074 (0.119)
=1 if HH head is educated		1.959 (5.774)		8.497** (3.387)		3.189 (3.611)
Female headed household		−7.591*** (2.656)		−9.554*** (2.884)		11.311* (6.369)
=1 if Eastern Region	2.385 (7.340)	1.164 (7.422)	4.900 (5.929)	3.741 (5.668)	n/a	n/a
=1 if Western Region	−13.600** (6.289)	−14.660** (6.210)	−9.634 (5.864)	−11.485** (5.521)		
=1 if Northern Region	−6.839 (5.901)	−7.842 (5.950)	−3.606 (5.243)	−6.060 (5.110)		
Season dummies?	No	No	Yes	Yes	Yes	Yes
HH fixed-effects?	No	No	No	No	Yes	Yes
Constant	26.302*** (5.709)	19.816** (8.697)	33.560*** (4.538)	33.284*** (7.027)	34.221*** (1.191)	39.587*** (8.579)
Observations	2190	2190	4422	4422	4422	4422
R^2^	0.033	0.049	0.027	0.052	0.033	0.039
Number of households					1244	1244

Notes: For simplicity, the first row shows the ITT estimates for all estimators with and without covariates. Columns (1) and (2) are the simple mean difference estimates ($\tau_{SMD}$); columns (3) and (4) are the DID treatment effect estimates $\left(\tau_{DID}\right)$, which is the ‘treated*post-intervention’ interaction term; and columns (5) and (6) are the treatment effects estimates from the FE estimator $\left(\tau_{FE}\right)$. Robust standard errors, clustered at the LC1 level, are shown in parentheses. ***p < 0.01, **p < 0.05, *p < 0.1.

**Table B.2c:** Treatment effects on share of area planted to improved varieties (sample without the treatment group contaminated observations).

Dependent variable: Share of improved maize area (%)	(1)	(2)	(3)	(4)	(5)	(6)
	SMD	SMD	DiD	DiD	FE	FE
Treatment effect (τ)	7.461** (3.629)	7.473* (3.718)	8.539* (4.847)	8.831* (4.874)	10.122** (4.071)	9.437** (4.146)
Household size		1.090*** (0.372)		0.630*** (0.223)		−1.152** (0.488)
Age of household head		−0.034 (0.071)		−0.139* (0.082)		−0.060 (0.116)
=1 if HH head is educated		2.556 (5.647)		8.712** (3.331)		2.767 (3.589)
Female headed household		−6.512** (2.966)		−9.021*** (2.962)		11.420* (6.439)
=1 if Eastern Region	3.647 (7.428)	2.397 (7.466)	5.541 (5.932)	4.405 (5.645)	n/a	n/a
=1 if Western Region	−13.990** (6.059)	−15.097** (6.018)	−9.769* (5.744)	−11.593** (5.409)		
=1 if Northern Region	−6.038 (5.809)	−7.219 (5.877)	−3.206 (5.194)	−5.692 (5.055)		
Season dummies?	No	No	Yes	Yes	Yes	Yes
HH fixed-effects?	No	No	No	No	Yes	Yes
Constant	26.660*** (5.670)	20.867** (8.539)	33.499*** (4.509)	33.599*** (7.039)	34.078*** (1.198)	40.005*** (8.759)
Observations	2223	2223	4455	4455	4455	4455
R^2^	0.033	0.047	0.026	0.052	0.031	0.036
Number of households					1244	1244

Notes: For simplicity, the first row shows the ITT estimates for all estimators with and without covariates. Columns (1) and (2) are the simple mean difference estimates ($\tau_{SMD}$); columns (3) and (4) are the DID treatment effect estimates $\left(\tau_{DID}\right)$, which is the ‘treated*post-intervention’ interaction term; and columns (5) and (6) are the treatment effects estimates from the FE estimator $\left(\tau_{FE}\right)$. Robust standard errors, clustered at the LC1 level, are shown in parentheses. ***p < 0.01, **p < 0.05, *p < 0.1.

**Table B.3:** Treatment effects on household storage capacity.

VARIABLES	(1)	(2)
	Ln (storage capacity)	Ln (storage capacity)
=1 if HH received a hermetic bag	0.039 (0.080)	0.029 (0.074)
Household size		0.059*** (0.014)
Age of household head		0.006** (0.002)
=1 if HH head is educated		0.360*** (0.079)
=1 if female headed household		−0.218*** (0.078)
=1 if Eastern Region	−0.248** (0.112)	−0.321*** (0.100)
=1 if Western Region	0.112 (0.121)	0.113 (0.100)
=1 if Northern Region	0.083 (0.139)	0.077 (0.134)
Season dummies?	Yes	Yes
Constant	6.573*** (0.068)	5.780*** (0.174)
Observations	3660	3660
R^2^	0.012	0.064

Notes: Robust standard errors, clustered at the LC1 level, are shown in parentheses.

***p < 0.01, **p < 0.05, *p < 0.1.

**Table C.1:** Treatment effects on household decisions to sell at harvest.

Dependent variable: 1 if HH sold maize at harvest	(1)	(2)	(3)	(4)	(5)	(6)
	SMD	SMD	DiD	DiD	FE	FE
Treatment effect (τ)	0.014 (0.041)	0.014 (0.042)	0.034 (0.038)	0.034 (0.038)	0.034 (0.039)	0.036 (0.039)
Household size		0.007** (0.003)		−0.001 (0.004)		−0.001 (0.006)
Age of household head		−0.003*** (0.001)		−0.003*** (0.001)		−0.000 (0.002)
=1 if HH head is educated		−0.033 (0.050)		−0.001 (0.025)		−0.035 (0.078)
Female headed household		−0.102** (0.041)		−0.062** (0.027)		−0.097 (0.089)
=1 if Eastern Region	−0.213*** (0.051)	−0.228*** (0.046)	−0.200*** (0.059)	−0.204*** (0.057)		
=1 if Western Region	0.025 (0.054)	−0.000 (0.052)	−0.032 (0.051)	−0.053 (0.048)		
=1 if Northern Region	−0.071* (0.040)	−0.097** (0.039)	−0.062 (0.043)	−0.084* (0.042)		
Season indicators	No	Yes	No	No	Yes	Yes
HH fixed effects?	No	No	No	No	Yes	Yes
Constant	0.545*** (0.033)	0.684*** (0.086)	0.738*** (0.039)	0.998*** (0.066)	0.763*** (0.017)	0.838*** (0.096)
Observations	2377	2377	4749	4749	4749	4749
R^2^	0.037	0.055	0.056	0.088	0.083	0.084
Number of households					1245	1245

Notes: For simplicity, the first row shows the ITT estimates for all estimators with and without covariates. Columns (1) and (2) are the simple mean difference estimates ($\tau_{SMD}$); columns (3) and (4) are the DID treatment effect estimates $\left(\tau_{DID}\right)$, which is the ‘treated*post-intervention’ interaction term; and columns (5) and (6) are the treatment effects estimates from the FE estimator $\left(\tau_{FE}\right)$. Robust standard errors, clustered at the LC1 level, are shown in parentheses. ***p < 0.01, **p < 0.05, *p < 0.1.

**Table C.2:** Treatment effects on storage at harvest for sales in the lean period.

Dependent variable:=1 if HH stored maize for sale at harvest	(1)	(2)	(3)	(4)	(5)	(6)
	SMD	SMD	DiD	DiD	FE	FE
Treatment effect (τ)	0.030 (0.023)	0.029 (0.024)	0.067** (0.030)	0.067** (0.030)	0.070** (0.031)	0.069** (0.030)
Household size		0.002 (0.004)		0.004 (0.003)		0.000 (0.005)
Age of household head		−0.000 (0.001)		−0.001 (0.001)		0.001 (0.002)
=1 if HH head is educated		0.023 (0.034)		0.043** (0.018)		0.007 (0.036)
Female headed household		−0.063* (0.034)		0.009 (0.028)		0.098** (0.037)
=1 if Eastern Region	−0.077*** (0.027)	−0.081*** (0.029)	−0.066*** (0.019)	−0.071*** (0.019)		
=1 if Western Region	0.099*** (0.027)	0.092*** (0.028)	0.092*** (0.023)	0.085*** (0.023)		
=1 if Northern Region (0/1)	0.105*** (0.030)	0.097*** (0.031)	0.070*** (0.021)	0.059*** (0.022)		
Season indicators	No	Yes	No	No	Yes	Yes
HH fixed effects?	No	No	No	No	Yes	Yes
Constant	0.182*** (0.018)	0.183*** (0.067)	0.153*** (0.014)	0.119** (0.053)	0.151*** (0.010)	0.094 (0.099)
Observations	2376	2376	4740	4740	4740	4740
R^2^	0.034	0.040	0.030	0.042	0.019	0.021
Number of households					1245	1245

Notes: For simplicity, the first row shows the ITT estimates for all estimators with and without covariates. Columns (1) and (2) are the simple mean difference estimates ($\tau_{SMD}$); columns (3) and (4) are the DID treatment effect estimates $\left(\tau_{DID}\right)$, which is the ‘treated*post-intervention’ interaction term; and columns (5) and (6) are the treatment effects estimates from the FE estimator $\left(\tau_{FE}\right)$. Robust standard errors, clustered at the LC1 level, are shown in parentheses. ***p < 0.01, **p < 0.05, *p < 0.1.

**Table C.3:** Treatment effects on maize yield.

Dependent variable: Maize yield (kg/ha)	(1)	(2)	(3)	(4)	(5)	(6)
	SMD	SMD	DiD	DiD	FE	FE
Treatment effect (τ)	88.871 (116.841)	88.856 (114.073)	172.546 (115.541)	178.541 (118.593)	94.495 (93.317)	90.766 (94.051)
Household size		19.172* (10.030)		22.632*** (7.791)		−0.366 (11.380)
Age of household head		−5.558* (3.209)		−5.063** (1.985)		−4.537* (2.312)
=1 if HH head is educated		−116.086 (108.131)		32.948 (66.048)		21.247 (138.264)
Female headed household		−375.977*** (79.089)		−245.574*** (66.112)		105.311 (195.721)
=1 if Eastern Region	−457.511*** (133.748)	−497.150*** (131.735)	−521.806*** (100.787)	−565.298*** (95.266)		
=1 if Western Region	204.193 (171.753)	133.768 (176.638)	105.843 (145.907)	48.493 (142.443)		
=1 if Northern Region	−253.063 (153.638)	−317.654* (162.919)	−263.550** (112.980)	−330.805*** (115.453)		
Season indicators	No	Yes	No	No	Yes	Yes
HH fixed effects?	No	No	No	No	Yes	Yes
Constant	1275.626*** (119.735)	1620.369*** (303.055)	1895.379*** (91.630)	2080.860*** (160.501)	1795.070*** (36.803)	1966.098*** (194.536)
Observations	2247	2247	4481	4481	4481	4481
R^2^	0.058	0.089	0.099	0.132	0.125	0.126
Number of households					1245	1245

Notes: For simplicity, the first row shows the ITT estimates for all estimators with and without covariates. Columns (1) and (2) are the simple mean difference estimates ($\tau_{SMD}$); columns (3) and (4) are the DID treatment effect estimates $\left(\tau_{DID}\right)$, which is the ‘treated*post-intervention’ interaction term; and columns (5) and (6) are the treatment effects estimates from the FE estimator $\left(\tau_{FE}\right)$. Robust standard errors, clustered at the LC1 level, are shown in parentheses. ***p < 0.01, **p < 0.05, *p < 0.1.

**Table C.4a:** Spillover effects on household planting improved maize varieties.

Dependent variable:=1 if HH planted improved maize	(1)	(2)	(3)	(4)	(5)	(6)
	SMD	SMD	DiD	DiD	FE	FE
Spillover effect (γ)	0.033	0.040	−0.036	−0.028	−0.025	−0.035
=1 if HH is in a DEMO village but not treated	(0.045)	(0.045)	(0.039)	(0.040)	(0.045)	(0.043)
Household size		0.008** (0.003)		0.004* (0.002)		−0.013** (0.005)
Age of household head		−0.000 (0.001)		−0.001 (0.001)		0.000 (0.001)
=1 if HH head is educated		0.019 (0.063)		0.072** (0.035)		0.013 (0.037)
Female headed household		−0.090*** (0.032)		−0.107*** (0.034)		0.124* (0.067)
=1 if Eastern Region	0.045 (0.086)	0.038 (0.085)	0.063 (0.061)	0.058 (0.056)		
=1 if Western Region	−0.130** (0.062)	−0.141** (0.062)	−0.102* (0.052)	−0.119** (0.048)		
=1 if Northern Region	−0.070 (0.062)	−0.080 (0.062)	−0.032 (0.051)	−0.056 (0.049)		
Season indicators	No	Yes	No	No	Yes	Yes
HH fixed effects?	No	No	No	No	Yes	Yes
Constant	0.257*** (0.063)	0.222** (0.100)	0.313*** (0.047)	0.337*** (0.078)	0.350*** (0.012)	0.394*** (0.081)
Observations	1804	1804	3591	3591	4039	4039
R^2^	0.029	0.043	0.032	0.057	0.035	0.041
Number of households					1243	1243

Notes: For simplicity, the first row shows the ITT estimates for all estimators with and without covariates. Columns (1) and (2) are the simple mean difference estimates (${\text{γ}}_{SMD}$); columns (3) and (4) are the DID treatment effect estimates $\left({\text{γ}}_{DID}\right)$, which is the ‘treated*post-intervention’ interaction term; and columns (5) and (6) are the treatment effects estimates from the FE estimator $\left({\text{γ}}_{FE}\right)$. Robust standard errors, clustered at the LC1 level, are shown in parentheses. ***p < 0.01, **p < 0.05, *p < 0.1.

**Table C.4b:** Spillover effects on share of area planted to improved varieties.

Dependent variable: Share of improved maize area (%)	(1)	(2)	(3)	(4)	(5)	(6)
	SMD	SMD	DiD	DiD	FE	FE
Spillover effect (γ)	3.834	4.497	−2.605	−1.833	−1.361	−2.311
=1 if HH is in a DEMO village but not treated	(4.357)	(4.351)	(3.729)	(3.786)	(4.345)	(4.124)
Household size		0.879*** (0.314)		0.422* (0.225)		−1.319** (0.540)
Age of household head		−0.018 (0.083)		−0.124 (0.090)		0.010 (0.097)
=1 if HH head is educated		0.998 (6.260)		6.609* (3.434)		1.382 (3.666)
Female headed household		−9.265*** (3.133)		−10.725*** (3.350)		11.437* (6.484)
=1 if Eastern Region	4.775 (8.122)	3.972 (8.115)	6.621 (5.848)	6.041 (5.385)		
=1 if Western Region	−12.215** (5.949)	−13.284** (5.962)	−9.760* (5.053)	−11.423** (4.676)		
=1 if Northern Region	−5.906 (5.939)	−6.770 (6.022)	−2.371 (4.924)	−4.730 (4.753)		
Season indicators	No	Yes	No	No	Yes	Yes
HH fixed effects?	No	No	No	No	Yes	Yes
Constant	24.363*** (5.941)	20.604** (9.734)	30.708*** (4.509)	33.045*** (7.676)	34.313*** (1.204)	39.306*** (7.855)
Observations						
R^2^	1804	1804	3590	3590	4038	4038
Number of households	0.028	0.043	0.033	0.056	0.035	0.041

Notes: For simplicity, the first row shows the ITT estimates for all estimators with and without covariates. Columns (1) and (2) are the simple mean difference estimates (${\text{γ}}_{SMD}$); columns (3) and (4) are the DID treatment effect estimates $\left({\text{γ}}_{DID}\right)$, which is the ‘treated*post-intervention’ interaction term; and columns (5) and (6) are the treatment effects estimates from the FE estimator $\left({\text{γ}}_{FE}\right)$. Robust standard errors, clustered at the LC1 level, are shown in parentheses. ***p < 0.01, **p < 0.05, *p < 0.1.

**Table C.4c:** Spillover effects on households using fertilizer.

Dependent variable:=1 if HH used fertilizer	(1)	(2)	(3)	(4)	(5)	(6)
	SMD	SMD	DiD	DiD	FE	FE
Spillover effect (γ)	−0.019	−0.008	−0.004	0.000	0.021	0.021
=1 if HH is in a DEMO village but not treated	(0.028)	(0.027)	(0.018)	(0.019)	(0.023)	(0.023)
Household size		0.002 (0.004)		0.005 (0.005)		0.001 (0.003)
Age of household head		−0.002*** (0.001)		−0.002** (0.001)		−0.000 (0.000)
=1 if HH head is educated		−0.049 (0.056)		−0.007 (0.031)		−0.063** (0.031)
Female headed household		−0.082*** (0.026)		−0.058*** (0.019)		0.006 (0.019)
=1 if Eastern Region	−0.072 (0.055)	−0.079 (0.049)	−0.065 (0.045)	−0.074 (0.044)		
=1 if Western Region	−0.194*** (0.034)	−0.213*** (0.035)	−0.183*** (0.033)	−0.200*** (0.036)		
=1 if Northern Region	−0.180*** (0.037)	−0.199*** (0.039)	−0.179*** (0.034)	−0.199*** (0.037)		
Season indicators	No	Yes	No	No	Yes	Yes
HH fixed effects?	No	No	No	No	Yes	Yes
Constant	0.241*** (0.034)	0.393*** (0.080)	0.206*** (0.033)	0.291*** (0.058)	0.119*** (0.007)	0.165*** (0.033)
Observations	1804	1804	3588	3588	4035	4035
R^2^	0.061	0.084	0.065	0.085	0.007	0.010
Number of households					1243	1243

Notes: For simplicity, the first row shows the ITT estimates for all estimators with and without covariates. Columns (1) and (2) are the simple mean difference estimates (${\text{γ}}_{SMD}$); columns (3) and (4) are the DID treatment effect estimates $\left({\text{γ}}_{DID}\right)$, which is the ‘treated*post-intervention’ interaction term; and columns (5) and (6) are the treatment effects estimates from the FE estimator $\left({\text{γ}}_{FE}\right)$. Robust standard errors, clustered at the LC1 level, are shown in parentheses. ***p < 0.01, **p < 0.05, *p < 0.1.

## Footnotes

1 For comparison, the local average treatment effect (LATE) estimates for main outcomes are shown later in this paper.

2 Dercon and Christiaensen (2011) examined risk in production technologies and welfare consequences on households when shocks resulted in a poor harvest. The risk in our context occurs during storage but the decision-making process is the same for either (pre-harvest or post-harvest) production risk.

3 One might ask why we did not examine impacts of our intervention on maize yields or output as one of the key impacts of our intervention. The reason is that yield or output is not a decision variable but rather an outcome variable, which is based on endogenous household decisions like seed, fertilizer and management decisions, along with exogenous factors like rainfall. Rainfall was low across Uganda in the season following our intervention, which also made it difficult for us to pick up a statistically significant impact of the hermetic bags on yields (see Appendix Table C.3 for the model of yields regressed on the hermetic bag treatment).

4 There are two types of losses: quantitative dry-weight loss and qualitative loss such as mold, discoloration, holes in maize from insect damage, etc. Like Kaminski & Christiaensen, we restrict our loss component to self-reported quantitative dry-weight loss.

5 Assuming the self-reported losses are true, the lower rates do not necessarily imply lack of post-harvest storage problems. Selling maize at low prices during the harvest period and buying at high prices in the lean period keeps households poor (e.g., see Stephens and Barrett, 2011).

6 We explore some of these measures in this paper and present more results in Appendix Table C.1, Table C.2.

7 LC1 is the lowest administrative unit in Uganda, and it sometimes comprises more than one village but we use LC1 and village interchangeably in this paper. The administrative units are at the central, district, sub-county, parish and LC1 levels, respectively.

8 For our power calculations, we used a minimum detectable effect (MDE) of 0.2 standard deviations—generally considered small MDE in practice (Beegle et al., 2017; Duflo et al., 2007)). Making a generous assumption about intra-cluster correlation coefficient (ICC) of zero (see Appendix Table A.3 for the actual ICC at baseline), at 80% power and 95% confidence level, a cluster size of 25 and 48 clusters should be sufficient to power our study given that 40% (P = 0.4) of the eligible households in DEMO villages were treated. In addition, we conducted a baseline survey that increased the number of observations used for analysis.

9 Besides, as a robustness check, we bound our estimates following Lee (2009) and present results for main outcomes in Appendix Table A.2. Our point estimates statistically lie within the estimated Lee bounds, further reducing concerns about attrition bias affecting the estimates.

10 We explored an additional specification to account for possible spillover effects on the main outcome variables. We compared non-treated households in DEMO villages with non-treated households in non-DEMO villages. We found no spillover effects (see Appendix Table C.4a, Table C.4b, Table C.4c). This is a logical finding because there is no reason to believe, and no direct theory of change to support the idea that households whose neighbors have a 100 kg capacity hermetic bag will cultivate a maize variety that is highly susceptible to pest attacks in storage. Only households with the technology can make that decision.

11 See Table A.3 in Appendix A for the ICC for the outcome variables.

12 We gave only one bag per household to treated households, which should minimize sharing or gifting of bags from treated to control households, and reduce unwanted crossovers/spillovers. However, if the majority of users in the exposed group—untreated households who received no bags in the DEMO villages (group 2 in Fig. 1)—got the bags from a treated neighbors or friends, the implication is that our estimated effects would be attenuated.

13 We thank one anonymous reviewer for bringing this to our attention.

14 We estimated LATE via Two-stage least squares (2SLS). Thus, the LATE estimates are compared to SMD estimates for each main outcome, respectively.

15 Given that there was no increase in quantity stored (Table 7) or yield effects (Appendix Table C.3) among treated households, one of the main effects of the intervention is improving food security through longer storage to improve consumption.

16 The metal silos evaluated in Gitonga et al. stored about 1 ton, which is 10 times the size of one hermetic bag evaluated in this study. This may explain the greater duration of storage in that study.

17 We estimated the ITT effects on on-farm storage post-harvest loss variable at the technology level rather than at the household level because the improved storage capacity is capped at 100 kg (1 bag) at the household level, which is about 6% and 24% of the average storage capacity and average quantity stored in the follow-up survey, respectively. Examining the treatment effects at the household level could create a ‘smearing effect’ where the impact of the hermetic bag on storage losses could be absorbed by the main (traditional) storage technology used. Indeed, we found a smearing effect when we estimated ITT at the household level. See Table A.5 in Appendix A for results at the household level.
